# Effective CRISPR interference of an endogenous gene via a single transgene in mice

**DOI:** 10.1038/s41598-019-53611-6

**Published:** 2019-11-21

**Authors:** Ryan S. MacLeod, Keisha M. Cawley, Igor Gubrij, Intawat Nookaew, Melda Onal, Charles A. O’Brien

**Affiliations:** 1 Center for Musculoskeletal Disease Research, University of Arkansas for Medical Sciences, Little Rock, 72205 AR USA; 2 Division of Endocrinology, Department of Internal Medicine, University of Arkansas for Medical Sciences, Little Rock, 72205 AR USA; 3 Department of Orthopaedic Surgery, University of Arkansas for Medical Sciences, Little Rock, 72205 AR USA; 4 Department of Bioinformatics, University of Arkansas for Medical Sciences, Little Rock, 72205 AR USA; 5 Department of Physiology and Biophysics, University of Arkansas for Medical Sciences, Little Rock, 72205 AR USA; 6 Central Arkansas Veterans Healthcare System, Little Rock, 72205 AR USA

**Keywords:** Gene expression, Genetics research

## Abstract

Drawbacks of conditional gene deletion in mice include the need for extensive breeding and, often, a lack of cell type-specificity. CRISPR interference (CRISPRi) is an alternative approach for loss-of-function studies that inhibits expression by guiding a transcriptional repressor to the transcription start-site of target genes. However, there has been limited exploration of CRISPRi in mice. We tested the effectiveness of a single CRISPRi transgene broadly expressing a single guide RNA and a catalytically dead Cas9 fused to the KRAB repressor domain to suppress a well-characterized target gene, *Tnfsf11*. The phenotype of CRISPRi transgenic mice was compared to mice with germline deletion of *Tnfsf11*, which are osteopetrotic and do not form lymph nodes. High transgene expression mimicked gene deletion, with failure of lymph node development and classic signs of osteopetrosis such as high bone mass and failure of tooth eruption. Mice with low transgene expression were normal and mice with medium expression displayed an intermediate phenotype. Transgene expression in tissues from these mice correlated inversely with *Tnfsf11* mRNA levels. These results demonstrate that a single CRISPRi transgene can effectively suppress a target gene in mice and suggest that this approach may be useful for cell type-specific loss-of-function studies.

## Introduction

Conditional gene deletion using the Cre-loxP recombination system has been widely used to interrogate gene function in mice, purportedly providing both cell type-specific and temporal control of gene inactivation^1^. Nonetheless, extensive use of this genetic tool over many years has revealed serious limitations of the system. The most important of these is that Cre-driver strains that were once thought to cause recombination in a single cell type have subsequently been shown to cause recombination in numerous additional cell types^2–6^. Because of this unwanted or off-target activity, it is often difficult to attribute the effects observed in a so-called cell type-specific loss-of-function (LOF) model to loss of the target gene in a single cell type. Another important limitation of conditional gene deletion is the need to perform multiple breeding cycles to obtain experimental mice. This limitation is notably increased if inactivation of multiple genes is desired.

Tools based on the bacterial CRISPR-Cas system are increasingly used for genetic modification^7–10^. This is due in part to the ease with which double-stranded DNA breaks can be introduced at or near a desired locus by altering the sequence of the single guide RNA (sgRNA) bound by the Cas9 nuclease^7,8^. The RNA-guided targeting of Cas proteins has also been used to control transcription of target genes without cutting DNA. The approach known as CRISPR interference (CRISPRi) inhibits gene expression by targeting a nuclease dead version of Cas9 (dCas9) to a region near the transcription start site (TSS)^11–13^. The degree of inhibition can be increased by fusing repressor domains, such as the Kruppel-associated box (KRAB) domain, to dCas9. Conversely, gene activation (CRISPRa) can be achieved by fusing transcriptional activation domains to dCas9^11,13,14^.

Certain aspects of CRISPRi make it a potentially appealing alternative to the Cre-loxP system for LOF studies in mice. For example, since the dCas9::KRAB protein can bind to both alleles of an endogenous gene, introduction of a DNA construct expressing both dCas9::KRAB and a sgRNA could potentially achieve LOF of the target gene using a single transgene. This would allow efficient production of LOF and control mice in a single generation, as compared to at least two generations using the bipartite Cre-loxP system. In addition, simultaneous expression of sgRNAs targeting different genes can repress expression of multiple target genes in the same cell or organism^13,15,16^. Thus, one can envision generation of a single transgenic mouse line in which multiple genes are repressed at the same time. Another potential advantage would be increased cell type-specificity. Whereas low levels of Cre recombinase activity are often sufficient to cause recombination in unwanted cell types, it is possible that low levels of dCas9::KRAB will have little or no ability to suppress target gene expression in unwanted cell types.

CRISPRi has been demonstrated in mice using viral vectors to introduce dCas9::KRAB and sgRNAs into somatic cells^15,17^. However, it remains unclear whether introduction of these components via a germline-integrated transgene can lead to effective and lasting target gene suppression. As a first step toward use of the CRISPRi system as an alternative to the Cre-loxP system, we tested the ability of a CRISPRi-transgene to repress transcription of a well-characterized gene (*Tnfsf11*) in mice.

## Results

In this study, we sought to determine if a single transgene is able to produce sufficient levels of dCas9::KRAB and sgRNA to effectively suppress a target gene in mice. To accomplish this, we aimed to globally suppress a target gene whose LOF phenotype is well characterized. We selected the *Tnfsf11* gene, which encodes the TNF-family cytokine known as receptor activator of NFκB ligand (RANKL)^18^. *Tnfsf11* is required for osteoclast formation in humans and mice^19,20^. Osteoclasts are large multinucleated cells of the myeloid lineage that are the only cells capable of resorbing mineralized cartilage and bone^21^. Bone resorption is required for several processes, including eruption of teeth from mandibular bone, shaping of the skull and long bones during growth, and remodeling of mineralized cartilage into cancellous bone during endochondral bone formation^19^. Consequently, mice lacking a functional *Tnfsf11* gene exhibit failure of tooth eruption, misshapen bones, and retention of mineralized cartilage beneath growth plates^19^. Collectively, this phenotype is known as osteopetrosis^22^. It is also important to note that *Tnfsf11*-null mice completely lack lymph nodes, but this is unrelated to the absence of osteoclasts^19^.

**Figure 1:**
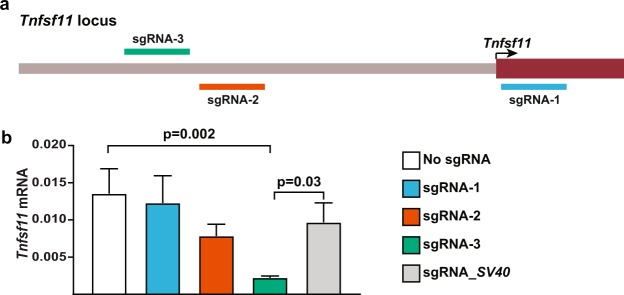
CRISPRi sgRNA selection and testing. (**a**) Diagram showing sgRNA position in relation to the transcription start site (TSS) of *Tnfsf11*. To repress transcription, the dCas9::KRAB/sgRNA complex was targeted to within 150 bp of the TSS on either the template or non-template strand of DNA. (**b**) Testing sgRNAs *in vitro* in UAMS-32 cells stably expressing dCas9::KRAB and either no sgRNA, one of the sgRNAs targeting the *Tnfsf11* TSS, or a sgRNA targeting the exogenous *SV40* promoter sequence as a control. This experiment was replicated at least once. One-way ANOVA with multiple comparisons between *Tnfsf11-*targeted sgRNAs and controls, *n* = 3 wells per cell pool.

We began by designing and testing several sgRNAs in a cell line that expresses *Tnfsf11*^23^. CRISPRi sgRNA sequences have optimal effects when targeted to within 150 base pairs (bp) of the TSS^11,14^. The DESKGEN CRISPR guide picker tool (Desktop Genetics) was used to select three sgRNAs targeting the 300 bp region centered on the *Tnfsf11* TSS based on high calculated on-target efficiencies and minimal off-target potential^11–14^ (Fig. 1a). The coding sequences for these sgRNAs were introduced into a lentiviral sgRNA expression vector and used to transduce a stromal cell line expressing the dCas9::KRAB fusion protein. A sgRNA targeting the SV40 TSS was used as a negative control. One of the three sgRNAs targeting *Tnfsf11* suppressed *Tnfsf11* mRNA abundance by approximately 80% relative to controls (Fig. 1b). This sgRNA was selected for use in the transgene.

**Figure 2:**
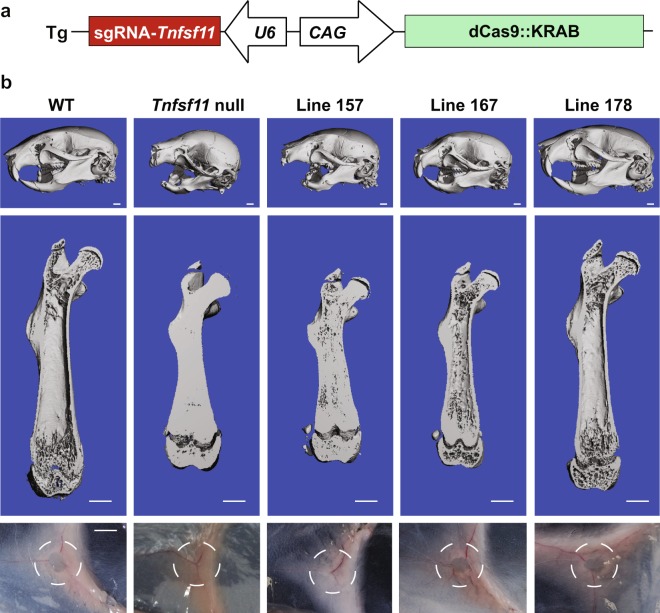
Transgene design and gross phenotype of *Tnfsf11* targeted CRISPRi mice. (**a**) Schematic representation of the transgene used to create *Tnfsf11* targeted CRISPRi mice. The dCas9::KRAB is under control of the CAG promoter which consists of the cytomegalovirus early enhancer; the promoter, first exon, and first intron of the chicken beta-actin gene; and the splice acceptor of the rabbit beta-globin gene. The sgRNA scaffold containing the targeting sequence of sgRNA-3 from the *in vitro* experiments was placed under the control of the mouse U6 small RNA promoter in the opposite direction of the CAG-driven dCas9::KRAB. (**b**) The top and middle images of each column are micro-CT generated images of the skull and right femur, respectively, of the indicated lines at five weeks of age. The images in the bottom row demonstrate the presence or lack of inguinal lymph nodes in each line. The dashed circle indicates the area of tissue where inguinal lymph node development normally occurs.

We designed the transgene to express the sgRNA as well as dCas9::KRAB (Fig. 2a). The dCas9::KRAB coding sequence was placed under the control of the broadly-expressed CAG promoter^24^ and the sgRNA coding sequence was placed under the control of the U6 promoter^25^ in a single DNA construct (Fig. 2a). This construct was then used to create mice by pronuclear injection of C57BL/6 zygotes. Seven mice harboring the transgene were identified by PCR amplification of tail DNA (Table 1). Of these, one mouse clearly lacked incisors (founder 176), suggestive of osteopetrosis (Table 1). Attempts were made to produce transgenic offspring from all mice. However, the mouse lacking incisors died before producing offspring, likely due to malnutrition. One founder that exhibited tooth eruption produced transgenic offspring lacking incisors (line 157), suggesting mosaicism in the founder. Another mouse did not transmit the transgene to offspring (Table 1). Preliminary microCT characterization of offspring from the five lines that produced offspring suggested that two of the lines displayed apparently normal bone mass, two had high bone mass, and the line lacking incisors was indeed osteopetrotic (Table 1).

**Table 1:** Characteristics of transgenic mouse lines.

Line/Founder	Tooth eruption	Skeletal phenotype
157	no	Osteopetrotic
160	yes	Normal
165	yes	Normal
167	yes	High bone mass
172	yes	High bone mass
174	yes	No germline transmission
176	no	Osteopetrotic; founder died before breeding
178	Yes	Normal

We selected three lines for additional characterization: the osteopetrotic line (157), one of the high bone mass lines (167), and one of the lines with apparently normal bone mass (178). MicroCT images of the skull of 5-week-old mice revealed that the skull from line 157 closely resembled that of *Tnfsf11*-null mice (Fig. 2b). It is important to note that the lack of tooth eruption in line 157 was partially penetrant in that, of the 8 mice analyzed from this line, 6 completely lacked tooth eruption, one displayed partial eruption of molar teeth, and one displayed partial eruption of incisors (Supplementary Fig. S1). Skulls from line 167 were smaller than wild type (WT) skulls but exhibited normal tooth eruption. Skulls from line 178 were indistinguishable from WT skulls (Fig. 2b). MicroCT images of femurs also showed similarities between line 157 and *Tnfsf11*-null mice, such as reduced length, compared to WT, and a marrow cavity filled with mineralized bone (Fig. 2b). However, the filling of the marrow cavity in line 157 was less complete than in *Tnfsf11*-null mice. The femur from line 167 also displayed increased trabecular bone in the marrow cavity and reduced length, but these changes were less pronounced than in line 157. The femur from line 178 appeared similar to that of WT mice (Fig. 2b). To examine a non-skeletal impact of *Tnfsf11* suppression, we examined whether lymph node formation was affected by the transgene. Inguinal lymph nodes were absent from line 157 and *Tnfsf11*-null mice but were present in the other two lines (Fig. 2b).

**Figure 3:**
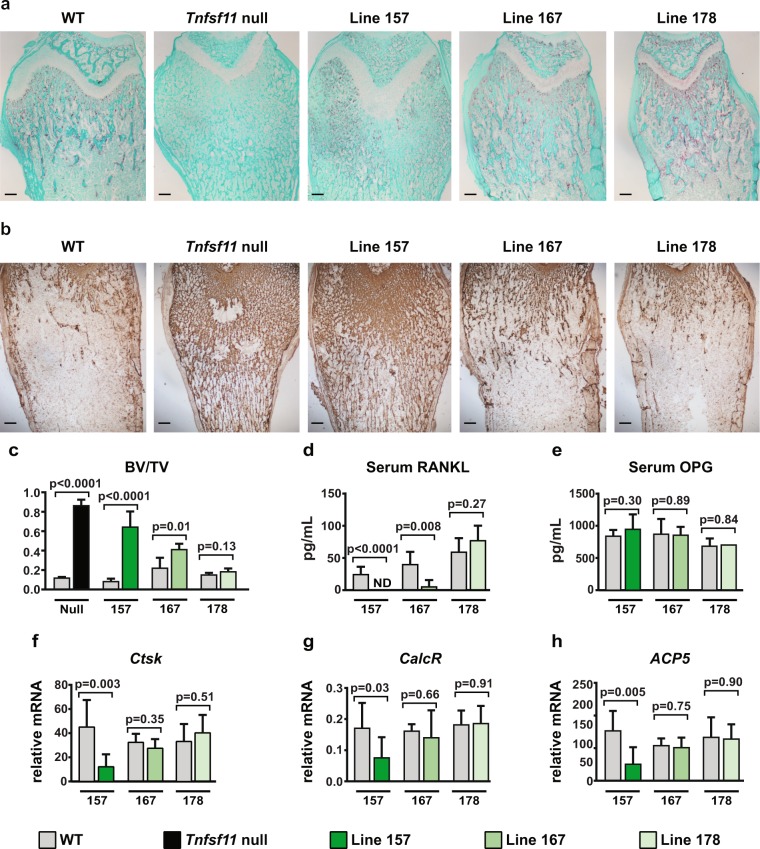
Bone phenotype of selected transgenic mouse lines. (**a**) Paraffin sections of the distal femurs of wild type (WT), *Tnfsf11* null, and the indicated transgenic mice stained for TRAPase activity. Osteoclasts are stained red. (**b**) Immunohistochemistry of paraffin sections of the distal femurs beneath the growth plate of WT, *Tnfsf11*-null, and the indicated transgenic lines for Type II collagen visualization. (**c**) Cancellous bone volume of right femurs from 5-week-old mice from line 157 WT (*n* = 6) and transgenic (TG) (*n* = 6), line 167 WT (*n* = 4) and TG (*n* = 5), and line 178 WT (*n* = 6) and TG (*n* = 3) mice. Both sexes were included. Transgenic compared to WT littermates of the same line using Student’s *t* test. (**d**) RANKL protein levels in serum of 5-week-old line 157 WT (*n* = 7) and TG (*n* = 7), line 167 WT (*n* = 4) and TG (*n* = 5), and line 178 WT (*n* = 6) and TG (*n* = 3) mice. Both sexes were included. ND, not detected. Transgenic compared to WT littermates of the same line using Student’s *t* test. (**e**) Osteoprotegerin (OPG) protein levels in serum of 5-week-old line 157 WT (*n* = 6) and TG (*n* = 7) mice, line 167 WT (*n* = 4) and TG (*n* = 5), and line 178 WT (*n* = 6) and TG (*n* = 2). Both sexes were included. Transgenic compared to WT littermates of the same line using Student’s *t* test. (**f**–**h**) Quantitative RT-qPCR analysis of *Cathepsin K*, *CalcR*, *and ACP5* (TRAP) mRNAs in L5 vertebrae from 5-week-old mice of line 157 WT (*n* = 6) and TG (*n* = 8), line 167 WT (*n* = 4) and TG (*n* = 5), and line 178 WT (*n* = 6) and TG (*n* = 3). Both sexes were included. Transgenic compared to WT littermates of the same line using Student’s *t* test.

As expected, histological staining for osteoclasts revealed a complete absence of these cells in *Tnfsf11*-null mice (Fig. 3a). Consistent with a lack of bone resorption, the bone marrow cavity of these mice retained large amounts of mineralized cartilage, as revealed by immunostaining for collagen II (Fig. 3b). Although bone sections from transgenic line 157 revealed the presence of some osteoclasts, their abundance was much lower compared with WT mice (Fig. 3a). In addition, the retention of calcified cartilage was similar to that observed in *Tnfsf11*-null mice (Fig. 3b). Transgenic line 167 exhibited a small decrease in osteoclast abundance with a mild increase in calcified cartilage retention, and osteoclast number in line 178 was similar to WT mice (Fig. 3b).

To quantify changes in bone mass, we measured trabecular bone volume by microCT in the three transgenic lines and compared each to their respective WT littermates. Consistent with the images in Fig. 2, line 157 had the highest bone volume of the three lines while line 167 had a smaller but still significant increase in bone volume (Fig. 3c). Bone volume in line 178 was similar to WT littermates (Fig. 3c). As an indirect measure of osteoclast number, we quantified *Cathepsin K*, *CalcR* (encoding calcitonin receptor), and *ACP5* (encoding tartrate-resistant acid phosphatase) mRNAs, which are highly expressed in osteoclasts, in RNA prepared from L5 vertebra. Line 157 had lower osteoclast-specific mRNAs than controls whereas the other two lines showed no changes (Fig. 3f–h).

Although RANKL is initially produced as a transmembrane protein, it can be cleaved by proteases to yield a soluble form that is functional and detectable in the circulation^26^. Therefore, we quantified soluble RANKL (sRANKL) in the serum of the three transgenic lines and found that it was not detectable in line 157 and significantly reduced in line 167 (Fig. 3d). Line 178 showed no changes in sRANKL (Fig. 3d). The actions of RANKL are opposed by a soluble decoy receptor known as osteoprotegerin (OPG), whose levels can be influenced by changes in RANKL production^27^. No changes in circulating OPG were observed in any of the three transgenic lines (Fig. 3e).

**Figure 4:**
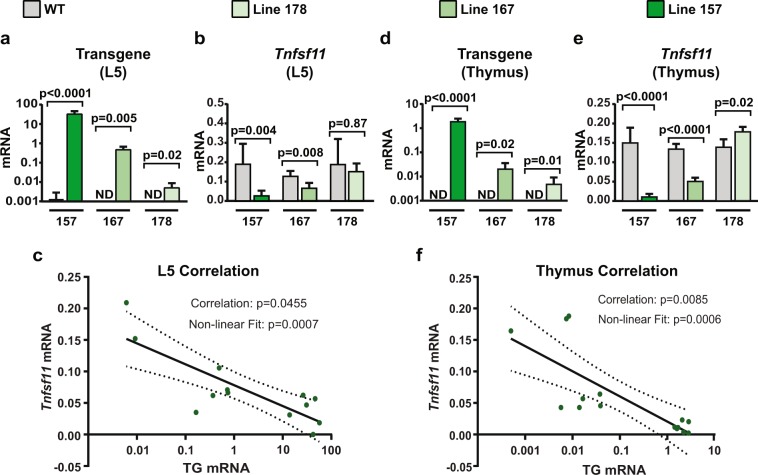
RNA expression in CRISPRi mouse lines. (**a**) Quantitative RT-qPCR analysis of transgene mRNA in L5 vertebrae from 5-week-old mice of line 157 WT (*n* = 6) and TG (*n* = 6), line 167 WT (*n* = 4) and TG (*n* = 5), and line 178 WT (*n* = 6) and TG (*n* = 3). Both sexes were included. Transgenic compared to WT littermates of the same line using Student’s *t* test. (**b**) Quantitative RT-qPCR analysis of *Tnfsf11* mRNA in L5 vertebrae from 5-week-old mice of line 157 WT (*n* = 6) and TG (*n* = 6), line 167 WT (*n* = 4) and TG (*n* = 5), and line 178 WT (*n* = 6) and TG (*n* = 3). Both sexes were included. Transgenic compared to WT littermates of the same line using Student’s *t* test. (**c**) Correlation analysis of the values shown in **a** and **b**. Correlation significance and line of best fit included. (**d**) Quantitative RT-qPCR analysis of transgene mRNA in thymus from 5-week-old mice of line 157 WT (*n* = 6) and TG (*n* = 7), line 167 WT (*n* = 4) and TG (*n* = 5), and line 178 WT (*n* = 5) and TG (*n* = 3). Both sexes were included Transgenics compared to WT littermates of the same line using Student’s *t* test. (**e**) Quantitative RT-qPCR analysis of *Tnfsf11* mRNA in thymus from 5-week-old mice of line 157 WT (*n* = 6) and TG (*n* = 7), line 167 WT (*n* = 4) and TG (*n* = 5), and line 178 WT (*n* = 5) and TG (*n* = 3). Both sexes were included. Transgenic compared to WT littermates of the same line using Student’s *t* test. (**f**) Correlation analysis of the values shown in (**d**,**e**). Correlation significance and line of best fit included.

The effectiveness of CRISPRi depends, in part, on the amount of dCas9::KRAB protein produced^28^. Therefore, we compared expression of the transgene among the three different lines. In bone, line 157 had the highest, line 167 had an intermediate, and line 178 had the lowest relative level of transgene expression (Fig. 4a). As anticipated, expression of *Tnfsf11* was inversely correlated with expression of the transgene in bone (Fig. 4b,c). A similar correlation between transgene and *Tnfsf11* expression was observed in the thymus (Fig. 4d–f). It is important to note that the suppression of *Tnfsf11* in line 157 was approximately 90%, which is similar to that observed in our *in vitro* test of the sgRNA.

**Figure 5:**
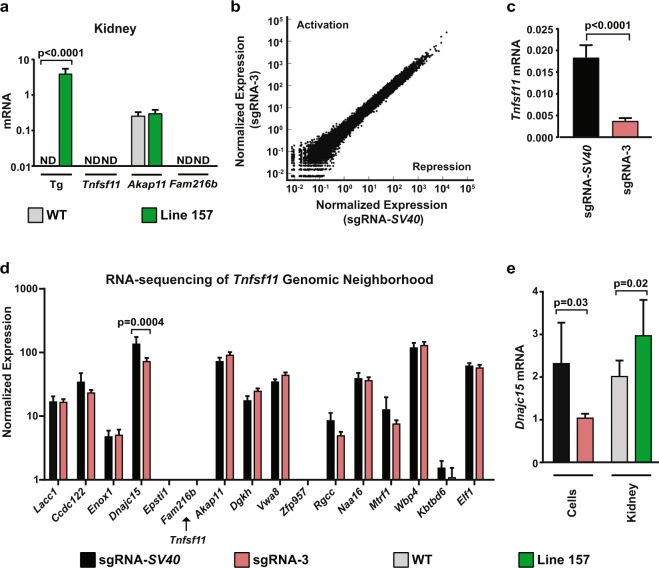
Specificity of CRISPRi. (**a**) Quantitative RT-qPCR analysis of transgene, *Tnfsf11*, *Akap11*, and *Fam216b* mRNA transcripts in kidney from 5-week-old mice of WT (*n* = 6) and TG (*n* = 6) mice from line 157. Both sexes were included. Transgenic compared to WT littermates of the same line using Student’s *t* test. (**b**) RNA-sequencing normalized expression patterns are plotted for UAMS-32 cells stably expressing dCas9::KRAB and either the sgRNA targeting *Tnfsf11* (sgRNA-3) or the exogenous *SV40* promoter sequence (sgRNA-*SV40*). No major transcriptome alterations occurred in the sgRNA-3 expressing cells. (**c**) Quantitative RT-qPCR analysis of *Tnfsf11* mRNA transcripts from the same samples used in the RNA-sequencing analysis for verification of *Tnfsf11* suppression. sgRNA-3 cells compared to sgRNA-*SV40* cells using Student’s *t* test. (**d**) Normalized expression values from the RNA-sequencing from UAMS-32 cells for the genes 1.5 Mb upstream (right side of plot) and downstream (left side of plot) from *Tnfsf11*. Relative location of *Tnfsf11* is indicated with the labeled arrow. Adjusted *p*-value displayed for genes with *p* < 0.05. (**e**) Quantitative RT-qPCR analysis of *Dnajc15* mRNA transcripts from UAMS-32 cells (sgRNA-3 versus sgRNA-*SV40*) and kidney from line 157 (n = 6) and their WT controls (n = 6). Both sexes were included. Comparisons were made using Student’s *t* test.

Lastly, we determined whether suppression of *Tnfsf11* by CRISPRi affected expression of nearby genes and the transcriptome as a whole. To do this, we measured expression of *Akap11*, which is upstream, and *Fam216b*, which is downstream of *Tnfsf11*, using the line expressing the highest level of the transgene. We used RNA from kidney, a tissue that is minimally affected by changes in *Tnfsf11*, to avoid detecting changes due to differences in cell type as opposed to changes in gene expression. *Fam216b* mRNA was undetectable in the kidney of either genotype, consistent with the previous observation that this gene is primarily expressed in the fallopian tube and parts of the lung and brain (Fig. 5a)^29^. *Akap11* mRNA was present at similar levels in WT and transgenic littermates (Fig. 5a). We also performed RNA-sequencing analysis using the cell lines in which we originally tested the effectiveness of the different sgRNAs (Fig. 1). There were no major changes in global transcription that would indicate off-target activity of the *Tnfsf11* targeted CRISPRi components (Fig. 5b). *Tnfsf11* transcripts were below the level of detection in the RNA-sequencing experiment (not shown). Therefore, we confirmed suppression of *Tnfsf11* by sgRNA-3 in the samples used for RNA-sequencing utilizing quantitative RT-PCR (Fig. 5c). To determine potential effects on additional nearby genes, we analyzed the normalized expression data from the RNA-sequencing experiment of the genes 1.5 Mb upstream and downstream from the *Tnfsf11* gene. Of these genes, only *Dnajc15* was significantly changed, exhibiting a 50% reduction in expression (Fig. 5d). We confirmed this suppression in the samples used for RNA-sequencing using quantitative RT-qPCR (Fig. 5e). In contrast, when we measured *Dnajc15* expression in kidney from Line 157 transgenic mice, we found that the relative expression increased in the transgenic mice (Fig. 5e). These contradictory results from our *in vitro* and *in vivo* RNA samples do not allow us to make a firm conclusion on the effects of sgRNA-3 on *Dnajc15* expression. However, the lack of concordance between sample types suggests that these differences most likely reflect normal variation in expression between samples rather than an effect of the CRISPRi components.

## Discussion

The results presented here demonstrate that CRISPRi via a single transgene is an effective tool for LOF studies in mice. We tested this idea using a transgene promoter that is active in a broad range of cell types. The reason for this approach, rather than starting with a cell type-specific promoter, was that we wanted to determine whether CRISPRi via transgenesis can provide effective and lasting gene suppression in different cell types. Our successful demonstration of this now sets the stage for determining whether a cell type-specific promoter can also produce sufficient amounts of dCas9::KRAB to achieve effective and lasting suppression and do so in a cell type-specific manner.

Generation of null alleles in mice is relatively straightforward using wild type Cas9 to create indels in early coding exons of a target gene. Thus, CRISPRi transgenesis is unlikely to be of broad benefit for global LOF studies. Instead, we anticipate that it will be useful for cell type-specific LOF based on the following rationale. Our results demonstrate that low levels of dCas9::KRAB expression do not suppress target gene transcription. Therefore, it is likely that low level expression of a cell type-specific transgene in unwanted cell types will not cause detectable suppression of the target gene in these unwanted cell types. In other words, the target gene should be suppressed only cell types expressing high levels of the transgene. This is in contrast to the Cre-loxP system, in which even very low levels of Cre expression can cause recombination of the target allele leading to complete LOF in unwanted cell types. Temporal control of gene deletion using a tamoxifen-regulated version of the Cre recombinase is a desirable aspect of the Cre-loxP system^30^. It is possible that temporal control of CRISPRi could be achieved by placing expression of dCas9::KRAB under the control of an inducible system, such as one of the tetracycline-regulated systems^31^. Importantly, the tetracycline-regulated system can be combined with a cell type-specific promoter to achieve both temporal and cell type-specific control of gene expression^32^.

We observed variation in the skeletal phenotype within the mouse line expressing a high level of dCas9::KRAB (line 157). While the majority of mice in this line exhibited a total lack of tooth eruption, two of the mice analyzed had either partial molar eruption or partial incisor eruption (Supplementary Fig. S1). This line also had variable amounts of bone marrow space in the femur, with many of the mice resembling the *Tnfsf11*-null mouse and others with partial formation of a marrow cavity (Supplementary Fig. S1). Nonetheless, lymph node development was abrogated in all transgenic mice analyzed from this line. These observations suggest that the level of *Tnfsf11* suppression in line 157 is sufficient to prevent lymph node organogenesis, but it is just at the threshold of suppression for complete absence of osteoclastogenesis and subsequent tooth eruption. Animal-to-animal variation in transgene expression may therefore explain the slight differences in the phenotype observed.

It is also important to note that even in the mice from line 157 with the strongest skeletal phenotype, the impact on the skeleton did not completely match that observed in *Tnfsf11*-null mice. The reasons for this are unclear but may be due to the relative expression level of the CAG promoter in different cell types. For example, *Tnfsf11* expression from several different cell types, including chondrocytes and osteocytes, contributes to osteoclast formation. Thus, residual *Tnfsf11* expression in one of these may have allowed low-level osteoclast formation to persist in the transgenic mice. Despite the residual expression of the target gene, the magnitude of the effect on osteoclast and lymph node development was sufficient to observe a major requirement of *Tnfsf11* for each of these processes. Based on this evidence, we think that pursuit of CRISPRi for cell type-specific LOF is worthwhile for situations in which a true cell type-specific Cre driver strain in not available.

Another potentially powerful utilization of CRISPRi transgenesis is that it may allow LOF studies in progenitors without causing LOF in cells descended from such progenitors. Since Cre-mediated recombination is irreversible, inactivation of a conditional allele in a progenitor using the Cre-loxP system necessarily leads to inactivation of the gene in all cells originating from the progenitor. Thus, it is often difficult to discern whether a phenotype is due to loss of the gene in the progenitor, its descendants, or the entire lineage. On the other hand, if a promoter can be identified that is active in a progenitor but not its descendants, then such a promoter can be used to express dCas9::KRAB and suppress target genes in the progenitor but not the progeny.

Transgenic expression of wild type Cas9 could also be used for cell type-specific LOF studies. However, a limitation of such an approach is that it would result in permanent modification of DNA, thus precluding progenitor-specific LOF. In addition, it is likely that use of intact Cas9 would result in generation of different indels in different cells, possibly even on sister chromosomes in the same cell, resulting in tissue mosaicism. One study using an inducible intact Cas9 cell line for LOF studies found that only 60% of cells had true gene knockout, and at least 30% of cells had in-frame indel mutations^28^. Such mosaicism may cause difficulty in interpretation of the resulting phenotype.

Introduction of CRISPRi components to mice via viral vectors has been reported earlier^15,17^ and is clearly more rapid than production of new transgenic mice. While this approach is useful for cell types easily targeted by viral vectors, such as neurons and hepatocytes^15,17^, it is not useful for many other cell types. For example, suppression of *Tnfsf11* in hypertrophic chondrocytes and osteocytes was required for the skeletal phenotype observed in the current study^33^. While precursors of these cell types can be transduced *in vitro*, it is not clear that either cell type can be successfully targeted by viral vectors *in vivo*. It is also important to note that introduction of CRISPRi components via viral vectors can induce an immune response^17,34^, which is avoided by transgenic CRISPRi.

A limitation of CRISPRi transgenesis is that a new transgenic mouse line needs to be created for each new gene to be suppressed. However, this limitation is somewhat balanced by the time saved in reducing the number of crosses needed to obtain experimental mice. Moreover, the increasing utilization of safe-harbor loci for introduction of transgenes should also facilitate the generation and characterization of novel transgenic lines^35^.

Another limitation of CRISPRi is that the factors governing the effectiveness of different sgRNAs are not fully known. Studies examining the effectiveness of hundreds to thousands of different sgRNAs have revealed some commonalities among effective sgRNAs^14,36^. The first is that a given sgRNA is more likely to repress transcription if it is positioned downstream from the transcription start site (TSS). The second is that targeting sites that are not occupied by nucleosomes appears to increase effectiveness. These guidelines are useful but it is often not possible to identify the exact TSS or nucleosome positioning for a given gene in a given cell type. This is the reason that genome-wide libraries of sgRNAs for CRISPRi usually include 10 or more sgRNAs targeting each gene^14^. Therefore, the most effective approach to identify sgRNAs that will effectively suppress a given target gene is to functionally test multiple sgRNAs targeting the region immediately surrounding the reported TSS in a cell culture model.

In summary, we have demonstrated herein that suppression of an endogenous gene via transgenic production of CRISPRi components in mice is sufficient to produce a phenotype similar to that produced by gene deletion. Continued exploration of this approach may allow LOF studies in mice that have not been possible due to lack of true cell type-specificity.

## Methods

### sgRNA *in silico* selection

Candidate sgRNAs were identified using the DESKGEN CRISPR guide picker cloud software (Desktop Genetics) by searching the region 150 bp upstream and downstream from the *Tnfsf11* TSS. The sgRNAs were screened based on off-target scores and predicted on-target efficiencies. An output from DESKGEN listing the position of each sgRNA, as well as on-target and off-target scores and locations, is presented in Supplementary Fig. S3. All sgRNAs conform to the nucleotide requirements for the U6 Pol III promoter and the *Streptococcus pyogenes* Cas9 PAM recognition sequence (NGG). Sequences for sgRNAs are as follows: sgRNA-1: GCCATCTCTCCCACGTCCC (PAM:GGG), sgRNA-2 GGAGGCCAGCTCTCTCCACG (PAM: AGG), sgRNA-3: GAGCCAATCAGCCTCCAGGA (PAM: GGG), and sgRNA_*SV40*: GAATAGCTCAGAGGCCGAGG (PAM: CGG).

### Lentiviral expression constructs

Lentiviral constructs for expression of dCas9::KRAB and the sgRNA cassette were obtained from Addgene. pHAGE EF1α dCas9-KRAB was a gift from Rene Maehr & Scot Wolfe (Addgene plasmid # 50919)^37^. pLX-sgRNA was a gift from Eric Lander & David Sabatini (Addgene plasmid # 50662)^38^. Target sequences for *Tnfsf11* and the SV40 exogenous promoter were introduced using overlap-extension PCR followed by restriction digest and ligation. For each sgRNA sequence, four primers were ordered from Integrated DNA Technologies: F1-AAACTCGAGTGTACAAAAAAGCAGGCTTTAAAG, R1- rc(GN_19_)GGTGTTTCGTCCTTTCC, F2- GN_19_GTTTTAGAGCTAGAAATAGCAA, and R2- AAAGCTAGCTAATGCCAACTTTGTACAAGAAAGCTG where GN_19_ is the target sequence of the sgRNA and rc(GN_19_) is the reverse complement of the sequence. The parent pLX-sgRNA vector was PCR amplified using F1 and R1 primers in one reaction and F2 and R2 primers in another. The products of these reactions were gel purified to remove parent pLX-sgRNA vector and combined in a third PCR amplification using primers F1 and R2. After gel purification of this reaction, the PCR products and pLX-sgRNA were digested using NheI and XhoI. Digests were ligated with the Quick Ligation Kit (New England Biolabs) and transformed into Stabl3 chemically competent cells (ThermoFisher).

### Virus production

HEK293T cells were maintained in Dulbecco’s Modified Eagle Medium (Gibco) with 10% fetal bovine serum (Atlanta Biologicals) and 1% penicillin-streptamycin-glutamine (Gibco). HEK293T cells were plated at 1 × 10^6^ cells/well in a six-well plate and incubated overnight. The next day, packaging plasmids and the lentiviral vector plasmid encoding either dCas9::KRAB or sgRNAs were transfected using TransIT-LT1 transfection reagent (Mirus) in DMEM lacking serum according to the manufacturer’s instructions. Virus was harvested 24 and 48 hours after transfection.

### Generation of stable dCas9::KRAB cell pools and co-expression with sgRNAs

UAMS-32 cells^23^ were maintained in alpha Minimum Essential Medium (Gibco) supplemented with 10% fetal bovine serum (Atlanta Biologicals) and 1% penicillin-streptamycin-glutamine (Gibco). UAMS-32 cells were plated at 1 × 10^5^ cells/well in a six-well plate and incubated overnight. Cells were incubated with lentivirus particles encoding dCas9::KRAB and 8 μg/mL hexadimethrine bromide overnight followed by a recovery period of 2 hours with αMEM and serum. After the recovery period, incubation with viral particles was repeated. Following transduction, cells were treated with 10 μg/mL puromycin for selection and maintenance of stable cell pools. For the experiments involving sgRNAs, UAMS-32 cells transduced with dCas9::KRAB were incubated with pLX-sgRNA lentivirus expressing the desired sgRNA sequence as described above. Following this transduction, cells were treated with 10 μg/mL blasticidin for selection and maintenance. UAMS-32 cells stably expressing dCas9::KRAB and one of the sgRNAs were plated in triplicate in a 12-well plate at a density of 7.5 × 10^4^ cells per well. UAMS-32 cells expressing dCas9::KRAB but no sgRNA were used as a control. RNA was isolated from cells after 48 hours using the RNeasy Plus Mini Kit according to the manufacturer’s instructions (Qiagen).

### Generation of CRISPRi mice

CRISPRi transgenic mice were generated using a DNA construct encoding the dCas9::KRAB fusion protein including a bovine growth hormone polyadenylation sequence inserted downstream from a 4.5 kb DNA fragment containing a cytomegalovirus enhancer, the chicken beta-actin promoter, and an artificial intron (CAG promoter)^39^. Upstream from the CAG promoter, a DNA fragment including the mouse U6 small RNA promoter and the gRNA-3 sequence and CRISPR sgRNA scaffold was introduced in the opposite orientation of the CAG promoter using a Geneblock (Integrated DNA Technologies). The sequence of the transgene was verified by direct sequencing and the annotated sequence is available in Supplementary Fig. S2. Transgenic mice were produced by microinjection of purified DNA into pronuclei of C57BL/6 mice. Founders were screened for the presence of the transgene using the following primers: forward 5′-ATCAATCGGCTCTCCGACTA-3′; reverse 5′-CCGTTGTGTGATCAGTTTGG-3′; product size 198 bp for transgenic and no band for WT. Breeding of founder mice and all subsequent offspring was performed with C57BL/6J mice. All mice were fed lab diet 8640 (Envigo), provided water ad libitum, and were maintained on a 12-hour light/dark cycle. All animal studies were carried out in accordance with the policies of, and with approval from, the Institutional Animal Care and Use Committee of the University of Arkansas for Medical Sciences.

### Gene expression analysis

RNA was isolated from the fifth lumbar vertebrae, thymus, and kidney by homogenization in Trizol Reagent (Life Technologies) according to the manufacturer’s instructions. RNA quantity and 260/280 ratio were determined using a Nanodrop instrument (Thermo Fisher Scientific), and RNA integrity was verified by resolution on 0.8% agarose gels. Five hundred nanograms of RNA isolated from *in vitro* cultures or mouse tissues was used to synthesize cDNA using the High-Capacity cDNA Reverse Transcription Kit (Applied Biosystems) according to the manufacturer’s directions. Transcript abundance in the cDNA was measured by quantitative PCR using TaqMan Universal PCR Master Mix (Life Technologies) and Taqman assays. PCR amplification and detection were carried out on an ABI StepOnePlus Real-Time PCR system (Applied Biosystems) as follows: 2-min holding stage at 50 C followed by a 10-min initial denaturation at 95 C, 40 cycles of amplification including denaturation at 95 C for 15 sec and annealing/extension at 60 C for 1 min. The following TaqMan assays from Life Technologies were used: *Cathespin K* (Mm00484036_m1), *Tnfsf11* (Mm00441908_m1), *Akap11* (Mm01313931_m1), *Fam216b* (Mm00617249_m1), the housekeeping gene *Mrps2* (Mm00475529_m1), and a custom assay that spans the artificial intron in the CAG promoter to measure CAG-transgene mRNA (for, 5′- GCCCCGGCTCTGACT-3′; rev, 5′- CAGCACAATAACCAGCACGTT-3′; probe, 5′-FAMACTCCCACAGCTCCTGNFQ-3′). Gene expression was calculated using the comparative threshold cycle (ΔCt) method by subtracting *Mrps2* Ct value from the transcript of interest Ct value^40^.

### Quantification of circulating RANKL and OPG protein

Blood was collected by retro-orbital bleeding into a microcentrifuge tube and allowed to clot at room temperature for 2 hours. Blood was then centrifuged at 2000 × *g* for 20 minutes to separate serum from cells. Soluble RANKL and OPG in serum was measured using mouse Quantikine kits (R&D Systems) according to the manual provided by the manufacturer. A five-parameter regression formula was used to calculate the sample concentrations from standard curves using GraphPad statistical software (Prism).

### Microcomputed tomography (microCT) analysis

MicroCT analysis was used to measure cortical and trabecular architecture of the femur. Femurs were dissected, cleaned of soft tissues, fixed in 10% Millonig’s formalin overnight, and transferred gradually from 70% to 100% ethanol. Dehydrated bones were then loaded into a 12.3-mm-diameter scanning tube containing 100% ethanol and scanned by a µCT (model µCT40, Scanco Biomedical, Bruttisellen, Switzerland) to generate three-dimensional voxel images (1024 × 1024 pixels) of bone samples. Medium resolution scans were performed (*E* = 55 kVp, *I* = 145 µA, integration time = 200 ms), and a Gaussian filter (sigma = 0.8, support = 1) was used to reduce signal noise.A threshold of 220 was applied to all scans. Measurements were made by drawing contours every 10–20 slices and using voxel counting for bone volume per tissue volume and sphere-filling distance transformation indices. Calibration of the machine and quality control scans were performed weekly using five density standards, and monthly spatial resolution verifications were performed using a tungsten wire rod. Calibration records were used for beam hardening correction.

### Histology

Left femurs were fixed in 10% Millonig’s formalin for 24 hours and transferred to 14.5% ethylenediaminetetraacetic acid for decalcification. Once decalcification was complete, femurs were transferred gradually from 70% to 100% ethanol. The decalcified bone samples were embedded in paraffin and 5 μm thick longitudinal sections were prepared. Sections were stained for tartrate-resistant acid phosphatase (TRAP) activity to detect osteoclasts and were counterstained with Fast Green.

### Immunohistochemistry

After removal of paraffin and rehydration of the sections, endogenous peroxidases were quenched in 3% H_2_O_2_ for 10 minutes. For antigen retrieval, sections were incubated at 37 C for 30 minutes in 0.1% hyaluronidase with 0.1% pronase in phosphate buffered saline (PBS). The sections were washed 3 times in 0.1% Tween-20 in PBS (PBS-T) and blocked for 30 minutes in 5% normal horse serum and 1% bovine serum albumin in PBS-T. Mouse monoclonal IgG1 COL2A1 antibody (II-II6B3, Developmental Studies Hybridoma Bank, University of Iowa) was diluted to 0.3 µg/mL in PBS with 5% normal horse serum and 1% bovine serum albumin and incubated with sections overnight in a humidified chamber at 4 C. This was followed by washing in PBS-T and incubation with biotinylated anti-mouse IgG for 30 minutes. After washing in PBS-T, samples were incubated 30 minutes in ABC reagent from the VectaStain Elite Mouse IgG Detection Kit (PK-6102, VectaStain, Vector Laboratories, Burlingame, CA) and washed thoroughly. Sections were developed with diaminobenzidine from VectaStain according to the kit instructions, dehydrated, and mounted.

### RNA-sequencing

The Center for Translational Pediatrics Research Genomics Core laboratory at the Arkansas Children’s Research Institute (ACRI) prepared sequencing libraries from RNA samples by use of the Lexogen (Lexogen, Greenland, NH) QuantSeq. 3′ mRNA-Seq Library Prep Kit FWD for sequencing on the Illumina NextSeq. 500 platform. The quality and quantity of input RNA was determined by use of the Advanced Analytical Fragment Analyzer (AATI) and Qubit (Life Technologies) instruments respectively. All samples had RQN (RNA quality number) values of 9.0 or above. Sequencing libraries were prepared by use of the Lexogen QuantSeq. 3′ mRNA-Seq Library Prep Kit FWD according to the manufacturer’s protocol. Briefly, total RNA (500 ng) was hybridized to an oligo-dT primer containing an Illumina-compatible sequence and reverse transcription was performed to synthesize the first strand cDNA. The RNA template was removed and second strand synthesis was performed using random primers that also contain Illumina compatible linker sequences. Second strand synthesis was followed by a magnetic bead purification and library amplification by PCR, which introduces sequences required for cluster generation and i7 indices for multiplexing on Illumina sequencers. Libraries were validated on the Fragment Analyzer for fragment size and quantified by use of a Qubit fluorometer. Equal amounts of each library were pooled for sequencing on the NextSeq. 500 platform using a high output flow cell to generate approximately 25–40 million 75 base single reads per sample.

### Bioinformatic analysis

The RNA-seq data processing and analysis were preprocessed following a previously reported workflow^41^. In brief, the raw fastq files were first assessed for quality and trimmed using SolexaQA++ software^42^ to obtain high quality reads. The high-quality reads were aligned using bwa software^43^. Only unique mapped reads were used to calculate expression count for individual samples. The count tables of all samples were normalized using the logCPM method^44^. The raw data files can be located in the Sequence Read Archive at the National Center for Biotechnology Information under the bioproject PRJNA541176.

### Statistics

One-way ANOVA was used to detect statistically significant differences between sgRNAs in the *in vitro* experiments. As each line of CRISPRi mice was created from different founders and were therefore genetically distinct, Student’s *t* test was used to detect statistically significant differences between transgene positive mice and their WT littermates. All *t* tests were two-sided and were performed after determining that the data were normally distributed and exhibited equal variances. Correlation tests for *Tnfsf11* and transgene mRNA and line of best fit were performed using a log_10_ scale axis for the transgene mRNA. All bar graphs show mean with standard deviation indicated with error bars. Sample sizes for animal experiments were selected based on previous experiments that had sufficient power to detect the expected effect sizes^33^. All individuals involved in analysis of samples were blinded to the group identity of the samples. All statistical tests were performed using GraphPad statistical software (Prism).

## Supplementary information

Supplementary Figures
