# Supplementary material for: Effective CRISPR interference of an endogenous gene via a single transgene in mice
Source: Sci Rep. 2019 Nov 21;9:17312. doi: 10.1038/s41598-019-53611-6 (PMC6872636; doi:10.1038/s41598-019-53611-6)
Supplement: Supplementary file 1 — Supplementary Figures [file 41598_2019_53611_MOESM1_ESM.pdf]

Supplementary information for: Effective CRISPR interference of an endogenous gene via a single transgene in mice

Ryan S. MacLeod, Keisha M. Cawley, Igor Gubrij, Intawat Nookaew, Melda Onal, and Charles A. O’Brien

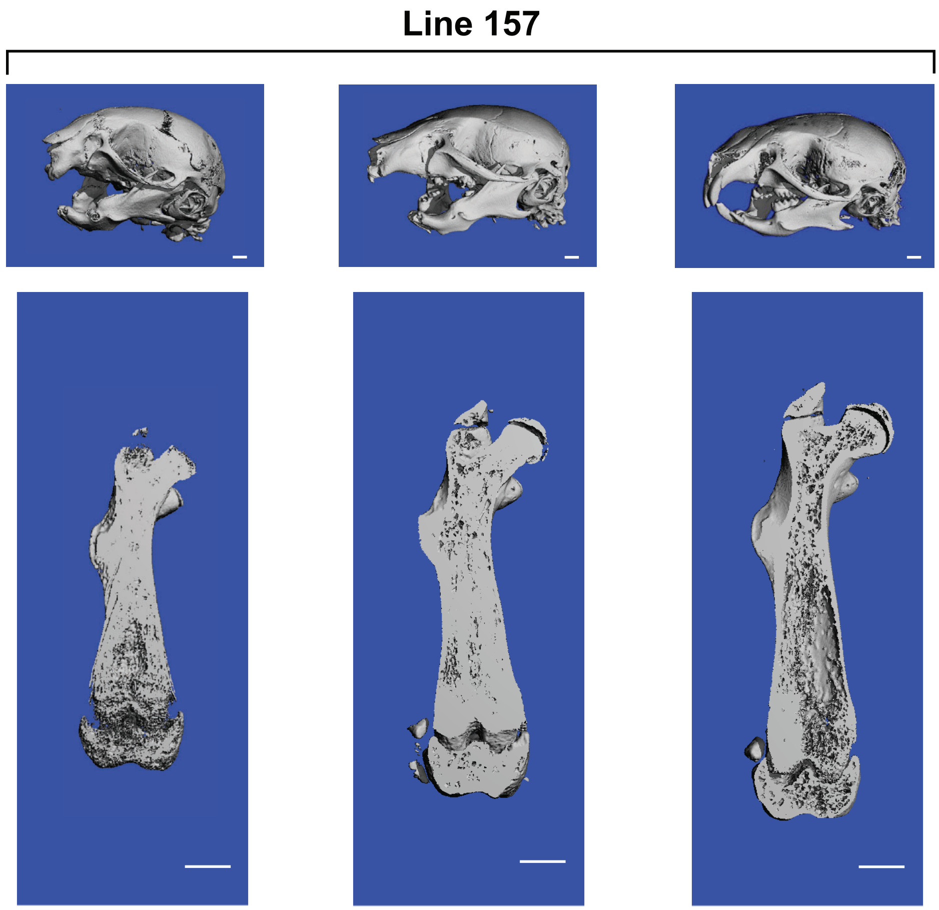

Supplementary Figure S1. Animal-to-animal variance within lines of CRISPRi mice. Micro-CT generated images of skulls and femurs of three different transgenic mice from line 157.

Supplementary Figure S1

Supplementary Figure S2. Diagram and annotated sequence of CRISPRi transgene.

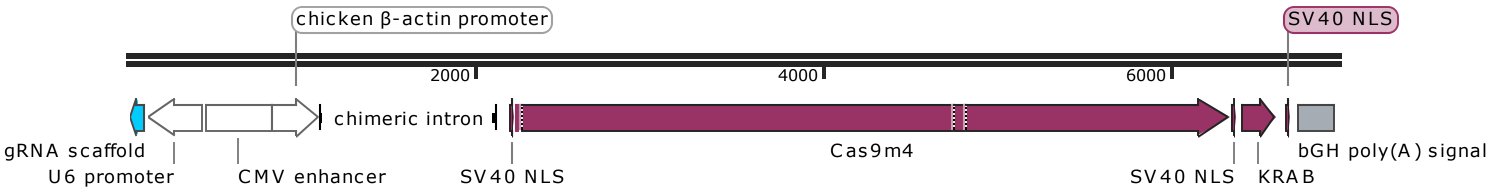

CAG-sg4 Transgene
6968 bp

5′ GCggccgcgcatAGTACAAAAAAAGCACCGACTCGGTGCCACTTTTTCAAGTTGATAACGGACTAGCCTTATTTTAACTT 80
3′ CGccggcgcgtaTCATGTTTTTTTCGTGGCTGAGCCACGGTGAAAAAGTTCAACTATTGCCTGATCGGAATAAAATTGAA
gRNA scaffold

GCTATTTCTAGCTCTAAAACTCCTGGAGGCTGATTGGCTCAAACAAGGCTTTTCTCCAAGGGATATTTATAGTCTCAAAA 160
CGATAAAGATCGAGATTTTGAGGACCTCCGACTAACCGAGTTTGTTCCGAAAAGAGGTTCCCTATAAATATCAGAGTTTT
gRNA scaffold U6 promoter

CACACAATTACTTTACAGTTAGGGTGAGTTTCCTTTTGTGCTGTTTTTTAAAATAATAATTTAGTATTTGTATCTCTTAT 240
GTGTGTTAATGAAATGTCAATCCCACTCAAAGGAAAACACGACAAAAAATTTTATTATTAAATCATAAACATAGAGAATA
U6 promoter

AGAAATCCAAGCCTATCATGTAAAATGTAGCTAGTATTAAAAAGAACAGATTATCTGTCTTTTATCGCACATTAAGCCTC 320
TCTTTAGGTTCGGATAGTACATTTTACATCGATCATAATTTTTCTTGTCTAATAGACAGAAAATAGCGTGTAATTCGGAG
U6 promoter

TATAGTTACTAGGAAATATTATATGCAAATTAACCGGGGCAGGGGAGTAGCCGAGCTTCTCCCACAAGTCTGTGCGAGGG 400
ATATCAATGATCCTTTATAATATACGTTTAATTGGCCCCGTCCCCTCATCGGCTCGAAGAGGGTGTTCAGACACGCTCCC
U6 promoter

GGCCGGCGCGGGCCTAGAGATGGCGGCGTCGGATCTATCTgcgatgcatATTTtcGACATTGATTATTGACTAGTTATTA 480
CCGGCCGCGCCCGGATCTCTACCGCCGCAGCCTAGATAGAcgctacgtaTAAAagCTGTAACTAATAACTGATCAATAAT
U6 promoter CMV enhancer

ATAGTAATCAATTACGGGGTCATTAGTTCATAGCCCATATATGGAGTTCCGCGTTACATAACTTACGGTAAATGGCCCGC 560
TATCATTAGTTAATGCCCCAGTAATCAAGTATCGGGTATATACCTCAAGGCGCAATGTATTGAATGCCATTTACCGGGCG
CMV enhancer

CTGGCTGACCGCCCAACGACCCCCGCCCATTGACGTCAATAATGACGTATGTTCCCATAGTAACGCCAATAGGGACTTTC 640
GACCGACTGGCGGGTTGCTGGGGGCGGGTAACTGCAGTTATTACTGCATACAAGGGTATCATTGCGGTTATCCCTGAAAG
CMV enhancer

CATTGACGTCAATGGGTGGAGTATTTACGGTAAACTGCCCACTTGGCAGTACATCAAGTGTATCATATGCCAAGTACGCC 720
GTAACTGCAGTTACCCACCTCATAAATGCCATTTGACGGGTGAACCGTCATGTAGTTCACATAGTATACGGTTCATGCGG
CMV enhancer

CCCTATTGACGTCAATGACGGTAAATGGCCCGCCTGGCATTATGCCCAGTACATGACCTTATGGGACTTTCCTACTTGGC 800
GGGATAACTGCAGTTACTGCCATTTACCGGGCGGACCGTAATACGGGTCATGTACTGGAATACCCTGAAAGGATGAACCG
CMV enhancer

AGTACATCTACGTATTAGTCATCGCTATTACCATGgtcgaggtgagccccacgttctgcttcactctccccatctccccc 880
TCATGTAGATGCATAATCAGTAGCGATAATGGTACcagctccactcggggtgcaagacgaagtgagaggggtagaggggg
CMV enhancer chicken β-actin promoter

```
ccctccccacccccaattttgtatttatttattttttaattattttgtgcagcgatgggggcggggggggggggggggcg   960
gggaggggtgggggttaaaacataaataaataaaaaattaataaaacacgtcgctacccccgcccccccccccccccccgc
                         chicken β-actin promoter >

cgcgccaggcggggcggggcggggcgaggggcggggcggggcgaggcggagaggtgcggcggcagccaatcagagcggcg  1040
gcgcggtccgccccgccccgccccgctccccgccccgccccgctccgcctctccacgccgccgtcggttagtctcgccgc
                         chicken β-actin promoter >

cgctccgaaagtttccttttatggcgaggcggcggcggcggcggccctataaaaagcgaagcgcgcggcgggcgggagtc  1120
gcgaggctttcaaaggaaaataccgctccgccgccgccgccgccgggatatttttcgcttcgcgcgccgcccgccctcag
                    chicken β-actin promoter >      chimeric intron

gctgcgcgctgccttcgccccgtgccccgctccgccgccgcctcgcgccgcccgccccggctctgactgaccgcgttact  1200
cgacgcgcgacggaagcggggcacggggcgaggcggcggcggagcgcggcgggcggggccgagactgactggcgcaatga
                         chimeric intron

cccacaggtgagcgggcgggacggcccttctcctccgggctgtaattagcgcttggtttaatgacggcttgtttcttttc  1280
gggtgtccactcgcccgccctgccgggaagaggaggcccgacattaatcgcgaaccaaattactgccgaacaaagaaaag
                         chimeric intron

tgtggctgcgtgaaagccttgaggggctccgggagggccctttgtgcggggggagcggctcggggggtgcgtgcgtgtgt  1360
acaccgacgcactttcggaactccccgaggccctcccgggaaacacgcccccctcgccgagccccccacgcacgcacaca
                         chimeric intron

gtgtgcgtggggagcgccgcgtgcggctccgcgctgcccggcggctgtgagcgctgcgggcgcggcgcggggctttgtgc  1440
cacacgcacccctcgcggcgcacgccgaggcgcgacgggccgccgacactcgcgacgcccgcgccgcgccccgaaacacg
                         chimeric intron

gctccgcagtgtgcgcgaggggagcgcggccgggggcggtgccccgcggtgcggggggggctgcgaggggaacaaaggct  1520
cgaggcgtcacacgcgctcccctcgcgccggcccccgccacggggcgccacgccccccccgacgctccccttgtttccga
                         chimeric intron

gcgtgcggggtgtgtgcgtgggggggtgagcagggggtgtgggcgcgtcggtcgggctgcaaccccccctgcacccccct  1600
cgcacgccccacacacgcacccccccactcgtcccccacacccgcgcagccagcccgacgttgggggggacgtgggggga
                         chimeric intron

ccccgagttgctgagcacggcccggcttcgggtgcggggctccgtacggggcgtggcgcggggctcgccgtgccgggcgg  1680
ggggctcaacgactcgtgccgggccgaagcccacgccccgaggcatgccccgcaccgcgccccgagcggcacggcccgcc
                         chimeric intron

ggggtggcggcaggtgggggtgccgggcggggcggggccgcctcgggccggggagggctcgggggaggggcgcggcggcc  1760
ccccaccgccgtccacccccacggcccgccccgccccggcggagcccggcccctcccgagccccctccccgcgccgccgg
                         chimeric intron
```

```
cccggagcgccggcggctgtcgaggcgcggcgagccgcagccattgccttttatggtaatcgtgcgagagggcgcaggga   1840
gggcctcgcggccgccgacagctccgcgccgctcggcgtcggtaacggaaaataccattagcacgctctcccgcgtccct
chimeric intron

cttcctttgtcccaaatctgtgcggagccgaaatctgggaggcgccgccgcaccccctctagcgggcgcggggcgaagcg   1920
gaaggaaacagggtttagacacgcctcggctttagaccctccgcggcggcgtgggggagatcgcccgcgccccgcttcgc
chimeric intron

gtgcggcgccggcaggaaggaaatgggcggggagggccttcgtgcgtcgccgcgccgccgtccccttctccctctccagc   2000
cacgccgcggccgtccttcctttacccgcccctcccggaagcacgcagcggcgcggcggcaggggaagagggagaggtcg
chimeric intron

ctcggggctgtccgcggggggacggctgccttcgggggggacggggcagggcggggttcggcttctggcgtgtgaccggc   2080
gagccccgacaggcgcccccctgccgacggaagccccccctgccccgtcccgccccaagccgaagaccgcacactggccg
chimeric intron

ggctctagagcctctgctaaccatgttcatgccttcttctttttcctacagctcctgggcaacgtgctggttattgtgct   2160
ccgagatctcggagacgattggtacaagtacggaagaagaaaaaggatgtcgaggacccgttgcacgaccaataacacga
chimeric intron

gtctcatcattttggcaaagaattAAATacgctgtGTTccggtgccaccATGCCCAAGAAGAAGAGGAAGGTGGGAAGGG   2240
cagagtagtaaaaccgtttcttaaTTTAtgcgacaCAAggccacggtggTACGGGTTCTTCTTCTCCTTCCACCCTTCCC
130
Pro Lys Lys Lys Arg Lys Val Gly Arg
SV40 NLS

GGATGGACAAGAAGTACTCCATTGGGCTCGCTATCGGCACAAACAGCGTCGGCTGGGCCGTCATTACGGACGAGTACAAG   2320
CCTACCTGTTCTTCATGAGGTAACCCGAGCGATAGCCGTGTTTGTCGCAGCCGACCCGGCAGTAATGCCTGCTCATGTTC
1 5 10 15 20 25
Gly Met Asp Lys Lys Tyr Ser Ile Gly Leu Ala Ile Gly Thr Asn Ser Val Gly Trp Ala Val Ile Thr Asp Glu Tyr Lys
Cas9m4

GTGCCGAGCAAAAAATTCAAAGTTCTGGGCAATACCGATCGCCACAGCATAAAGAAGAACCTCATTGGCGCCCTCCTGTT   2400
CACGGCTCGTTTTTTAAGTTTCAAGACCCGTTATGGCTAGCGGTGTCGTATTTCTTCTTGGAGTAACCGCGGGAGGACAA
30 35 40 45 50
Val Pro Ser Lys Lys Phe Lys Val Leu Gly Asn Thr Asp Arg His Ser Ile Lys Lys Asn Leu Ile Gly Ala Leu Leu Phe
Cas9m4

CGACTCCGGGGAGACGGCCGAAGCCACGCGGCTCAAAAGAACAGCACGGCGCAGATATACCCGCAGAAAGAATCGGATCT   2480
GCTGAGGCCCCTCTGCCGGCTTCGGTGCGCCGAGTTTTCTTGTCGTGCCGCGTCTATATGGGCGTCTTTCTTAGCCTAGA
55 60 65 70 75
Asp Ser Gly Glu Thr Ala Glu Ala Thr Arg Leu Lys Arg Thr Ala Arg Arg Arg Tyr Thr Arg Arg Lys Asn Arg Ile
Cas9m4

GCTACCTGCAGGAGATCTTTAGTAATGAGATGGCTAAGGTGGATGACTCTTTCTTCCATAGGCTGGAGGAGTCCTTTTTG   2560
CGATGGACGTCCTCTAGAAATCATTACTCTACCGATTCCACCTACTGAGAAAGAAGGTATCCGACCTCCTCAGGAAAAAC
80 85 90 95 100 105
Cys Tyr Leu Gln Glu Ile Phe Ser Asn Glu Met Ala Lys Val Asp Asp Ser Phe Phe His Arg Leu Glu Glu Ser Phe Leu
Cas9m4
```

GTGGAGGAGGATAAAAAGCACGAGCGCCACCCAATCTTTGGCAATATCGTGGACGAGGTGGCGTACCATGAAAAGTACCC 2640
CACCTCCTCCTATTTTTCGTGCTCGCGGTGGGTTAGAAACCGTTATAGCACCTGCTCCACCGCATGGTACTTTTCATGGG

110 115 120 125 130
Val Glu Glu Asp Lys Lys His Glu Arg His Pro Ile Phe Gly Asn Ile Val Asp Glu Val Ala Tyr His Glu Lys Tyr Pro

Cas9m4

AACCATATATCATCTGAGGAAGAAGCTTGTAGACAGTACTGATAAGGCTGACTTGCGGTTGATCTATCTCGCGCTGGCGC 2720
TTGGTATATAGTAGACTCCTTCTTCGAACATCTGTCATGACTATTCCGACTGAACGCCAACTAGATAGAGCGCGACCGCG

135 140 145 150 155
Thr Ile Tyr His Leu Arg Lys Lys Leu Val Asp Ser Thr Asp Lys Ala Asp Leu Arg Leu Ile Tyr Leu Ala Leu Ala

Cas9m4

ATATGATCAAATTTCGGGGACACTTCCTCATCGAGGGGGACCTGAACCCAGACAACAGCGATGTCGACAAACTCTTTATC 2800
TATACTAGTTTAAAGCCCCTGTGAAGGAGTAGCTCCCCCTGGACTTGGGTCTGTTGTCGCTACAGCTGTTTGAGAAATAG

160 165 170 175 180 185
His Met Ile Lys Phe Arg Gly His Phe Leu Ile Glu Gly Asp Leu Asn Pro Asp Asn Ser Asp Val Asp Lys Leu Phe Ile

Cas9m4

CAACTGGTTCAGACTTACAATCAGCTTTTCGAAGAGAACCCGATCAACGCATCCGGAGTTGACGCCAAAGCAATCCTGAG 2880
GTTGACCAAGTCTGAATGTTAGTCGAAAAGCTTCTCTTGGGCTAGTTGCGTAGGCCTCAACTGCGGTTTCGTTAGGACTC

190 195 200 205 210
Gln Leu Val Gln Thr Tyr Asn Gln Leu Phe Glu Glu Asn Pro Ile Asn Ala Ser Gly Val Asp Ala Lys Ala Ile Leu Ser

Cas9m4

CGCTAGGCTGTCCAAATCCCGGCGGCTCGAAAACCTCATCGCACAGCTCCCTGGGGAGAAGAAGAACGGCCTGTTTGGTA 2960
GCGATCCGACAGGTTTAGGGCCGCCGAGCTTTTGGAGTAGCGTGTCGAGGGACCCCTCTTCTTCTTGCCGGACAAACCAT

215 220 225 230 235
Ala Arg Leu Ser Lys Ser Arg Arg Leu Glu Asn Leu Ile Ala Gln Leu Pro Gly Glu Lys Lys Asn Gly Leu Phe Gly

Cas9m4

ATCTTATCGCCCTGTCACTCGGGCTGACCCCCAACTTTAAATCTAACTTCGACCTGGCCGAAGATGCCAAGCTTCAACTG 3040
TAGAATAGCGGGACAGTGAGCCCGACTGGGGGTTGAAATTTAGATTGAAGCTGGACCGGCTTCTACGGTTCGAAGTTGAC

240 245 250 255 260 265
Asn Leu Ile Ala Leu Ser Leu Gly Leu Thr Pro Asn Phe Lys Ser Asn Phe Asp Leu Ala Glu Asp Ala Lys Leu Gln Leu

Cas9m4

AGCAAAGACACCTACGATGATGATCTCGACAATCTGCTGGCCCAGATCGGCGACCAGTACGCAGACCTTTTTTTGGCGGC 3120
TCGTTTCTGTGGATGCTACTACTAGAGCTGTTAGACGACCGGGTCTAGCCGCTGGTCATGCGTCTGGAAAAAAACCGCCG

270 275 280 285 290
Ser Lys Asp Thr Tyr Asp Asp Asp Leu Asp Asn Leu Leu Ala Gln Ile Gly Asp Gln Tyr Ala Asp Leu Phe Leu Ala Ala

Cas9m4

AAAGAACCTGTCAGACGCCATTCTGCTGAGTGATATTCTGCGAGTGAACACGGAGATCACCAAAGCTCCGCTGAGCGCTA 3200
TTTCTTGGACAGTCTGCGGTAAGACGACTCACTATAAGACGCTCACTTGTGCCTCTAGTGGTTTCGAGGCGACTCGCGAT

295 300 305 310 315
Lys Asn Leu Ser Asp Ala Ile Leu Leu Ser Asp Ile Leu Arg Val Asn Thr Glu Ile Thr Lys Ala Pro Leu Ser Ala

Cas9m4

GTATGATCAAGCGCTATGATGAGCACCACCAAGACTTGACTTTGCTGAAGGCCCTTGTCAGACAGCAACTGCCTGAGAAG 3280
CATACTAGTTCGCGATACTACTCGTGGTGGTTCTGAACTGAAACGACTTCCGGGAACAGTCTGTCGTTGACGGACTCTTC

320 325 330 335 340 345
Ser Met Ile Lys Arg Tyr Asp Glu His His Gln Asp Leu Thr Leu Leu Lys Ala Leu Val Arg Gln Gln Leu Pro Glu Lys

Cas9m4

TACAAGGAAATTTTCTTCGATCAGTCTAAAAATGGCTACGCCGGATACATTGACGGCGGAGCAAGCCAGGAGGAATTTTA 3360
ATGTTCCTTTAAAAGAAGCTAGTCAGATTTTTACCGATGCGGCCTATGTAACTGCCGCCTCGTTCGGTCCTCCTTAAAAT

350 355 360 365 370
Tyr Lys Glu Ile Phe Phe Asp Gln Ser Lys Asn Gly Tyr Ala Gly Tyr Ile Asp Gly Gly Ala Ser Gln Glu Glu Phe Tyr

Cas9m4

CAAATTTATTAAGCCCATCTTGGAAAAAATGGACGGCACCGAGGAGCTGCTGGTAAAGCTTAACAGAGAAGATCTGTTGC 3440
GTTTAAATAATTCGGGTAGAACCTTTTTTACCTGCCGTGGCTCCTCGACGACCATTTCGAATTGTCTCTTCTAGACAACG

375 380 385 390 395
Lys Phe Ile Lys Pro Ile Leu Glu Lys Met Asp Gly Thr Glu Glu Leu Leu Val Lys Leu Asn Arg Glu Asp Leu Leu

Cas9m4

GCAAACAGCGCACTTTCGACAATGGAAGCATCCCCCACCAGATTCACCTGGGCGAACTGCACGCTATCCTCAGGCGGCAA 3520
CGTTTGTCGCGTGAAAGCTGTTACCTTCGTAGGGGGTGGTCTAAGTGGACCCGCTTGACGTGCGATAGGAGTCCGCCGTT

400 405 410 415 420 425
Arg Lys Gln Arg Thr Phe Asp Asn Gly Ser Ile Pro His Gln Ile His Leu Gly Glu Leu His Ala Ile Leu Arg Arg Gln

Cas9m4

GAGGATTTCTACCCCTTTTTGAAAGATAACAGGGAAAAGATTGAGAAAATCCTCACATTTCGGATACCCTACTATGTAGG 3600
CTCCTAAAGATGGGGAAAAACTTTCTATTGTCCCTTTTCTAACTCTTTTAGGAGTGTAAAGCCTATGGGATGATACATCC

430 435 440 445 450
Glu Asp Phe Tyr Pro Phe Leu Lys Asp Asn Arg Glu Lys Ile Glu Lys Ile Leu Thr Phe Arg Ile Pro Tyr Tyr Val Gly

Cas9m4

CCCCCTCGCCCGGGGAAATTCCAGATTCGCGTGGATGACTCGCAAATCAGAAGAGACCATCACTCCCTGGAACTTCGAGG 3680
GGGGGAGCGGGCCCCTTTAAGGTCTAAGCGCACCTACTGAGCGTTTAGTCTTCTCTGGTAGTGAGGGACCTTGAAGCTCC

455 460 465 470 475
Pro Leu Ala Arg Gly Asn Ser Arg Phe Ala Trp Met Thr Arg Lys Ser Glu Glu Thr Ile Thr Pro Trp Asn Phe Glu

Cas9m4

AAGTCGTGGATAAGGGGGCCTCTGCCCAGTCCTTCATCGAAAGGATGACTAACTTTGATAAAAATCTGCCTAACGAAAAG 3760
TTCAGCACCTATTCCCCCGGAGACGGGTCAGGAAGTAGCTTTCCTACTGATTGAAACTATTTTTAGACGGATTGCTTTTC

480 485 490 495 500 505
Glu Val Val Asp Lys Gly Ala Ser Ala Gln Ser Phe Ile Glu Arg Met Thr Asn Phe Asp Lys Asn Leu Pro Asn Glu Lys

Cas9m4

GTGCTTCCTAAACACTCTCTGCTGTACGAGTACTTCACAGTTTATAACGAGCTCACCAAGGTCAAATACGTCACAGAAGG 3840
CACGAAGGATTTGTGAGAGACGACATGCTCATGAAGTGTCAAATATTGCTCGAGTGGTTCCAGTTTATGCAGTGTCTTCC

510 515 520 525 530
Val Leu Pro Lys His Ser Leu Leu Tyr Glu Tyr Phe Thr Val Tyr Asn Glu Leu Thr Lys Val Lys Tyr Val Thr Glu Gly

Cas9m4

GATGAGAAAGCCAGCATTCCTGTCTGGAGAGCAGAAGAAAGCTATCGTGGACCTCCTCTTCAAGACGAACCGGAAAGTTA 3920
CTACTCTTTCGGTCGTAAGGACAGACCTCTCGTCTTCTTTCGATAGCACCTGGAGGAGAAGTTCTGCTTGGCCTTTCAAT

535 540 545 550 555
Met Arg Lys Pro Ala Phe Leu Ser Gly Glu Gln Lys Lys Ala Ile Val Asp Leu Leu Phe Lys Thr Asn Arg Lys Val

Cas9m4

CCGTGAAACAGCTCAAAGAgGACTATTTCAAAAAGATTGAATGTTTCGACTCTGTTGAAATCAGCGGAGTGGAGGATCGC 4000
GGCACTTTGTCGAGTTTCTcCTGATAAAGTTTTTCTAACTTACAAAGCTGAGACAACTTTAGTCGCCTCACCTCCTAGCG

560 565 570 575 580 585
Thr Val Lys Gln Leu Lys Glu Asp Tyr Phe Lys Lys Ile Glu Cys Phe Asp Ser Val Glu Ile Ser Gly Val Glu Asp Arg

Cas9m4

TTCAACGCATCCCTGGGAACGTATCACGATCTCCTGAAAATCATTAAAGACAAGGACTTCCTGGACAATGAGGAGAACGA 4080
AAGTTGCGTAGGGACCCTTGCATAGTGCTAGAGGACTTTTAGTAATTTCTGTTCCTGAAGGACCTGTTACTCCTCTTGCT

590 595 600 605 610
Phe Asn Ala Ser Leu Gly Thr Tyr His Asp Leu Leu Lys Ile Ile Lys Asp Lys Asp Phe Leu Asp Asn Glu Glu Asn Glu

Cas9m4

GGACATTCTTGAGGACATTGTCCTCACCCTTACGTTGTTTGAAGATAGGGAGATGATTGAAGAACGCTTGAAAACTTACG 4160
CCTGTAAGAACTCCTGTAACAGGAGTGGGAATGCAACAAACTTCTATCCCTCTACTAACTTCTTGCGAACTTTTGAATGC

615 620 625 630 635
Asp Ile Leu Glu Asp Ile Val Leu Thr Leu Thr Leu Phe Glu Asp Arg Glu Met Ile Glu Glu Arg Leu Lys Thr Tyr

Cas9m4

CTCATCTCTTCGACGACAAAGTCATGAAACAGCTCAAGAGGCGCCGATATACAGGATGGGGGCGGCTGTCAAGAAAACTG 4240
GAGTAGAGAAGCTGCTGTTTCAGTACTTTGTCGAGTTCTCCGCGGCTATATGTCCTACCCCCGCCGACAGTTCTTTTGAC

640 645 650 655 660 665
Ala His Leu Phe Asp Asp Lys Val Met Lys Gln Leu Lys Arg Arg Arg Tyr Thr Gly Trp Gly Arg Leu Ser Arg Lys Leu

Cas9m4

ATCAATGGGATCCGAGACAAGCAGAGTGGAAAGACAATCCTGGATTTTCTTAAGTCCGATGGATTTGCCAACCGGAACTT 4320
TAGTTACCCTAGGCTCTGTTCGTCTCACCTTTCTGTTAGGACCTAAAAGAATTCAGGCTACCTAAACGGTTGGCCTTGAA

670 675 680 685 690
Ile Asn Gly Ile Arg Asp Lys Gln Ser Gly Lys Thr Ile Leu Asp Phe Leu Lys Ser Asp Gly Phe Ala Asn Arg Asn Phe

Cas9m4

CATGCAGTTGATCCATGATGACTCTCTCACCTTTAAGGAGGACATCCAGAAAGCACAAGTTTCTGGCCAGGGGGACAGTC 4400
GTACGTCAACTAGGTACTACTGAGAGAGTGGAAATTCCTCCTGTAGGTCTTTCGTGTTCAAAGACCGGTCCCCCTGTCAG

695 700 705 710 715
Met Gln Leu Ile His Asp Asp Ser Leu Thr Phe Lys Glu Asp Ile Gln Lys Ala Gln Val Ser Gly Gln Gly Asp Ser

Cas9m4

TcCACGAGCACATCGCTAATCTTGCAGGTAGCCCAGCTATCAAAAAGGGAATACTGCAGACCGTTAAGGTCGTGGATGAA 4480
AgGTGCTCGTGTAGCGATTAGAACGTCCATCGGGTCGATAGTTTTTCCCTTATGACGTCTGGCAATTCCAGCACCTACTT

720 725 730 735 740 745
Leu His Glu His Ile Ala Asn Leu Ala Gly Ser Pro Ala Ile Lys Lys Gly Ile Leu Gln Thr Val Lys Val Val Asp Glu

Cas9m4

CTCGTCAAAGTAATGGGAAGGCATAAGCCCGAGAATATCGTTATCGAGATGGCCCGAGAGAACCAAACTACCCAGAAGGG 4560
GAGCAGTTTCATTACCCTTCCGTATTCGGGCTCTTATAGCAATAGCTCTACCGGGCTCTCTTGGTTTGATGGGTCTTCCC
750 755 760 765 770
Leu Val Lys Val Met Gly Arg His Lys Pro Glu Asn Ile Val Ile Glu Met Ala Arg Glu Asn Gln Thr Thr Gln Lys Gly
Cas9m4

ACAGAAGAACAGTAGGGAAAGGATGAAGAGGATTGAAGAGGGTATAAAAGAACTGGGGTCCCAAATCCTTAAGGAACACC 4640
TGTCTTCTTGTCATCCCTTTCCTACTTCTCCTAACTTCTCCCATATTTTCTTGACCCCAGGGTTTAGGAATTCCTTGTGG
775 780 785 790 795
Gln Lys Asn Ser Arg Glu Arg Met Lys Arg Ile Glu Glu Gly Ile Lys Glu Leu Gly Ser Gln Ile Leu Lys Glu His
Cas9m4

CAGTTGAAAACACCCAGCTTCAGAATGAGAAGCTCTACCTGTACTACCTGCAGAACGGCAGGGACATGTACGTGGATCAG 4720
GTCAACTTTTGTGGGTCGAAGTCTTACTCTTCGAGATGGACATGATGGACGTCTTGCCGTCCCTGTACATGCACCTAGTC
800 805 810 815 820 825
Pro Val Glu Asn Thr Gln Leu Gln Asn Glu Lys Leu Tyr Leu Tyr Tyr Leu Gln Asn Gly Arg Asp Met Tyr Val Asp Gln
Cas9m4

GAACTGGACATCAATCGGCTCTCCGACTACGACGTGGCTGCTATCGTGCCCCAGTCTTTTCTCAAAGATGATTCTATTGA 4800
CTTGACCTGTAGTTAGCCGAGAGGCTGATGCTGCACCGACGATAGCACGGGGTCAGAAAAGAGTTTCTACTAAGATAACT
830 835 840 845 850
Glu Leu Asp Ile Asn Arg Leu Ser Asp Tyr Asp Val Ala Ala Ile Val Pro Gln Ser Phe Leu Lys Asp Asp Ser Ile Asp
Cas9m4

TAATAAAGTGTTGACAAGATCCGATAAAgcTAGAGGGAAGAGTGATAACGTCCCCTCAGAAGAAGTTGTCAAGAAAATGA 4880
ATTATTTCACAACTGTTCTAGGCTATTTcgATCTCCCTTCTCACTATTGCAGGGGAGTCTTCTTCAACAGTTCTTTTACT
855 860 865 870 875
Asn Lys Val Leu Thr Arg Ser Asp Lys Ala Arg Gly Lys Ser Asp Asn Val Pro Ser Glu Glu Val Val Lys Lys Met
Cas9m4

AAAATTATTGGCGGCAGCTGCTGAACGCCAAACTGATCACACAACGGAAGTTCGATAATCTGACTAAGGCTGAACGAGGT 4960
TTTTAATAACCGCCGTCGACGACTTGCGGTTTGACTAGTGTGTTGCCTTCAAGCTATTAGACTGATTCCGACTTGCTCCA
880 885 890 895 900 905
Lys Asn Tyr Trp Arg Gln Leu Leu Asn Ala Lys Leu Ile Thr Gln Arg Lys Phe Asp Asn Leu Thr Lys Ala Glu Arg Gly
Cas9m4

GGCCTGTCTGAGTTGGATAAAGCCGGCTTCATCAAAAGGCAGCTTGTTGAGACACGCCAGATCACCAAGCACGTGGCCCA 5040
CCGGACAGACTCAACCTATTTCGGCCGAAGTAGTTTTCCGTCGAACAACTCTGTGCGGTCTAGTGGTTCGTGCACCGGGT
910 915 920 925 930
Gly Leu Ser Glu Leu Asp Lys Ala Gly Phe Ile Lys Arg Gln Leu Val Glu Thr Arg Gln Ile Thr Lys His Val Ala Gln
Cas9m4

AATTCTCGATTCACGCATGAACACCAAGTACGATGAAAATGACAAACTGATTCGAGAGGTGAAAGTTATTACTCTGAAGT 5120
TTAAGAGCTAAGTGCGTACTTGTGGTTCATGCTACTTTTACTGTTTGACTAAGCTCTCCACTTTCAATAATGAGACTTCA
935 940 945 950 955
Ile Leu Asp Ser Arg Met Asn Thr Lys Tyr Asp Glu Asn Asp Lys Leu Ile Arg Glu Val Lys Val Ile Thr Leu Lys
Cas9m4

CTAAGCTGGTCTCAGATTTCAGAAAGGACTTTCAGTTTTATAAGGTGAGAGAGATCAACAATTACCACCATGCGCATGAT 5200
GATTCGACCAGAGTCTAAAGTCTTTCCTGAAAGTCAAAATATTCCACTCTCTCTAGTTGTTAATGGTGGTACGCGTACTA

960 965 970 975 980 985
Ser Lys Leu Val Ser Asp Phe Arg Lys Asp Phe Gln Phe Tyr Lys Val Arg Glu Ile Asn Asn Tyr His His Ala His Asp

Cas9m4

GCCTACCTGAATGCAGTGGTAGGCACTGCACTTATCAAAAAATATCCCAAGCTTGAATCTGAATTTGTTTACGGAGACTA 5280
CGGATGGACTTACGTCACCATCCGTGACGTGAATAGTTTTTTATAGGGTTCGAACTTAGACTTAAACAAATGCCTCTGAT

990 995 1000 1005 1010
Ala Tyr Leu Asn Ala Val Val Gly Thr Ala Leu Ile Lys Lys Tyr Pro Lys Leu Glu Ser Glu Phe Val Tyr Gly Asp Tyr

Cas9m4

TAAAGTGTACGATGTTAGGAAAATGATCGCAAAGTCTGAGCAGGAAATAGGCAAGGCCACCGCTAAGTACTTCTTTTACA 5360
ATTTCACATGCTACAATCCTTTTACTAGCGTTTCAGACTCGTCCTTTATCCGTTCCGGTGGCGATTCATGAAGAAAATGT

1015 1020 1025 1030 1035
Lys Val Tyr Asp Val Arg Lys Met Ile Ala Lys Ser Glu Gln Glu Ile Gly Lys Ala Thr Ala Lys Tyr Phe Phe Tyr

Cas9m4

GCAATATTATGAATTTTTTCAAGACCGAGATTACACTGGCCAATGGAGAGATTCGGAAGCGACCACTTATCGAAACAAAC 5440
CGTTATAATACTTAAAAAAGTTCTGGCTCTAATGTGACCGGTTACCTCTCTAAGCCTTCGCTGGTGAATAGCTTTGTTTG

1040 1045 1050 1055 1060 1065
Ser Asn Ile Met Asn Phe Phe Lys Thr Glu Ile Thr Leu Ala Asn Gly Glu Ile Arg Lys Arg Pro Leu Ile Glu Thr Asn

Cas9m4

GGAGAAACAGGAGAAATCGTGTGGGACAAGGGTAGGGATTTCGCGACAGTCCGGAAGGTCCTGTCCATGCCGCAGGTGAA 5520
CCTCTTTGTCCTCTTTAGCACACCCTGTTCCCATCCCTAAAGCGCTGTCAGGCCTTCCAGGACAGGTACGGCGTCCACTT

1070 1075 1080 1085 1090
Gly Glu Thr Gly Glu Ile Val Trp Asp Lys Gly Arg Asp Phe Ala Thr Val Arg Lys Val Leu Ser Met Pro Gln Val Asn

Cas9m4

CATCGTTAAAAAGACCGAAGTACAGACCGGAGGCTTCTCCAAGGAAAGTATCCTCCCGAAAAGGAACAGCGACAAGCTGA 5600
GTAGCAATTTTTCTGGCTTCATGTCTGGCCTCCGAAGAGGTTCCTTTCATAGGAGGGCTTTTCCTTGTCGCTGTTCGACT

1095 1100 1105 1110 1115
Ile Val Lys Lys Thr Glu Val Gln Thr Gly Gly Phe Ser Lys Glu Ser Ile Leu Pro Lys Arg Asn Ser Asp Lys Leu

Cas9m4

TCGCACGCAAAAAAGATTGGGACCCCAAGAAATACGGCGGATTCGATTCTCCTACAGTCGCTTACAGTGTACTGGTTGTG 5680
AGCGTGCGTTTTTTCTAACCCTGGGGTTCTTTATGCCGCCTAAGCTAAGAGGATGTCAGCGAATGTCACATGACCAACAC

1120 1125 1130 1135 1140 1145
Ile Ala Arg Lys Lys Asp Trp Asp Pro Lys Lys Tyr Gly Gly Phe Asp Ser Pro Thr Val Ala Tyr Ser Val Leu Val Val

Cas9m4

GCCAAAGTGGAGAAAGGGAAGTCTAAAAAACTCAAAAGCGTCAAGGAACTGCTGGGCATCACAATCATGGAGCGATCAAG 5760
CGGTTTCACCTCTTTCCCTTCAGATTTTTTGAGTTTTCGCAGTTCCTTGACGACCCGTAGTGTTAGTACCTCGCTAGTTC

1150 1155 1160 1165 1170
Ala Lys Val Glu Lys Gly Lys Ser Lys Lys Leu Lys Ser Val Lys Glu Leu Leu Gly Ile Thr Ile Met Glu Arg Ser Ser

Cas9m4

CTTCGAAAAAAACCCCATCGACTTTCTCGAGGCGAAAGGATATAAAGAGGTCAAAAAAGACCTCATCATTAAGCTTCCCA 5840

GAAGCTTTTTTTGGGGTAGCTGAAAGAGCTCCGCTTTCCTATATTTCTCCAGTTTTTTCTGGAGTAGTAATTCGAAGGGT

1175 1180 1185 1190 1195

Phe Glu Lys Asn Pro Ile Asp Phe Leu Glu Ala Lys Gly Tyr Lys Glu Val Lys Lys Asp Leu Ile Ile Lys Leu Pro

Cas9m4

AGTACTCTCTCTTTGAGCTTGAAAACGGCCGGAAACGAATGCTCGCTAGTGCGGGCGAGCTGCAGAAAGGTAACGAGCTG 5920

TCATGAGAGAGAAACTCGAACTTTTGCCGGCCTTTGCTTACGAGCGATCACGCCCGCTCGACGTCTTTCCATTGCTCGAC

1200 1205 1210 1215 1220 1225

Lys Tyr Ser Leu Phe Glu Leu Glu Asn Gly Arg Lys Arg Met Leu Ala Ser Ala Gly Glu Leu Gln Lys Gly Asn Glu Leu

Cas9m4

GCACTGCCCTCTAAATACGTTAATTTCTTGTATCTGGCCAGCCACTATGAAAAGCTCAAAGGGTCTCCCGAAGATAATGA 6000

CGTGACGGGAGATTTATGCAATTAAAGAACATAGACCGGTCGGTGATACTTTTCGAGTTTCCCAGAGGGCTTCTATTACT

1230 1235 1240 1245 1250

Ala Leu Pro Ser Lys Tyr Val Asn Phe Leu Tyr Leu Ala Ser His Tyr Glu Lys Leu Lys Gly Ser Pro Glu Asp Asn Glu

Cas9m4

GCAGAAGCAGCTGTTCGTGGAACAACACAAACACTACCTTGATGAGATCATCGAGCAAATAAGCGAATTCTCCAAAAGAG 6080

CGTCTTCGTCGACAAGCACCTTGTTGTGTTTGTGATGGAACTACTCTAGTAGCTCGTTTATTCGCTTAAGAGGTTTTCTC

1255 1260 1265 1270 1275

Gln Lys Gln Leu Phe Val Glu Gln His Lys His Tyr Leu Asp Glu Ile Ile Glu Gln Ile Ser Glu Phe Ser Lys Arg

Cas9m4

TGATCCTCGCCGACGCTAACCTCGATAAGGTGCTTTCTGCTTACAATAAGCACAGGGATAAGCCCATCAGGGAGCAGGCA 6160

ACTAGGAGCGGCTGCGATTGGAGCTATTCCACGAAAGACGAATGTTATTCGTGTCCCTATTCGGGTAGTCCCTCGTCCGT

1280 1285 1290 1295 1300 1305

Val Ile Leu Ala Asp Ala Asn Leu Asp Lys Val Leu Ser Ala Tyr Asn Lys His Arg Asp Lys Pro Ile Arg Glu Gln Ala

Cas9m4

GAAAACATTATCCACTTGTTTACTCTGACCAACTTGGGCGCGCCTGCAGCCTTCAAGTACTTCGACACCACCATAGACAG 6240

CTTTTGTAATAGGTGAACAAATGAGACTGGTTGAACCCGCGCGGACGTCGGAAGTTCATGAAGCTGTGGTGGTATCTGTC

1310 1315 1320 1325 1330

Glu Asn Ile Ile His Leu Phe Thr Leu Thr Asn Leu Gly Ala Pro Ala Ala Phe Lys Tyr Phe Asp Thr Thr Ile Asp Arg

Cas9m4

AAAGCGGTACACCTCTACAAAGGAGGTCCTGGACGCCACACTGATTCATCAGTCAATTACGGGGCTCTATGAAACAAGAA 6320

TTTCGCCATGTGGAGATGTTTCCTCCAGGACCTGCGGTGTGACTAAGTAGTCAGTTAATGCCCCGAGATACTTTGTTCTT

1335 1340 1345 1350 1355

Lys Arg Tyr Thr Ser Thr Lys Glu Val Leu Asp Ala Thr Leu Ile His Gln Ser Ile Thr Gly Leu Tyr Glu Thr Arg

Cas9m4

TCGACCTCTCTCAGCTCGGTGGAGACAGCAGGGCTGACCCCAAGAAGAAGAGGAAGGTGGctagCGATGCTAAGTCACTG 6400

AGCTGGAGAGAGTCGAGCCACCTCTGTCGTCCCGACTGGGGTTCTTCTTCTCCTTCCACCgatcGCTACGATTCAGTGAC

1360 1365 130

Ile Asp Leu Ser Gln Leu Gly Gly Asp Ser Arg Ala Asp Pro Lys Lys Lys Arg Lys Val Ala Ser Asp Ala Lys Ser Leu

Cas9m4 SV40 NLS (in frame with SV40 NLS)

```
ACTGCCTGGTCCCGGACACTGGTGACCTTCAAGGATGTGTTTGTGGACTTCACCAGGGAGGAGTGGAAGCTGCTGGACAC   6480
TGACGGACCAGGGCCTGTGACCACTGGAAGTTCCTACACAAACACCTGAAGTGGTCCCTCCTCACCTTCGACGACCTGTG
                    15                  20                  25                  30
Thr Ala Trp Ser Arg Thr Leu Val Thr Phe Lys Asp Val Phe Val Asp Phe Thr Arg Glu Glu Trp Lys Leu Leu Asp Thr
KRAB

TGCTCAGCAGATCCTGTACAGAAATGTGATGCTGGAGAACTATAAGAACCTGGTTTCCTTGGGTTATCAGCTTACTAAGC   6560
ACGAGTCGTCTAGGACATGTCTTTACACTACGACCTCTTGATATTCTTGGACCAAAGGAACCCAATAGTCGAATGATTCG
    35                  40                  45                  50                  55
Ala Gln Gln Ile Leu Tyr Arg Asn Val Met Leu Glu Asn Tyr Lys Asn Leu Val Ser Leu Gly Tyr Gln Leu Thr Lys
KRAB

CAGATGTGATCCTCCGGTTGGAGAAGGGAGAAGAGCCCTGGCTGGTGGAGAGAGAAATTCACCAAGAGACCCATCCTGAT   6640
GTCTACACTAGGAGGCCAACCTCTTCCCTCTTCTCGGGACCGACCACCTCTCTCTTTAAGTGGTTCTCTGGGTAGGACTA
60                  65                  70                  75
Pro Asp Val Ile Leu Arg Leu Glu Lys Gly Glu Glu Pro Trp Leu Val Glu Arg Glu Ile His Gln Glu Thr His Pro Asp
KRAB                                              (in frame with KRAB)

TCAGAGACTGCATTTGAAATCAAATCATCAGTTCCGAAAAAGAAACGCAAAGttGctagCtgaTAAGaattcctagagct   6720
AGTCTCTGACGTAAACTTTAGTTTAGTAGTCAAGGCTTTTTCTTTGCGTTTCaaCgatcGactATTCttaaggatctcga
                                                  130
Ser Glu Thr Ala Phe Glu Ile Lys Ser Ser Val Pro Lys Lys Lys Arg Lys Val Ala Ser *
(in frame with KRAB)                         SV40 NLS

cgctgatcagcctcgactgtgccttctagttgccagccatctgttgtttgcccctcccccgtgccttccttgaccctgga   6800
gcgactagtcggagctgacacggaagatcaacggtcggtagacaacaaacggggagggggcacggaaggaactgggacct
bGH poly(A) signal

aggtgccactcccactgtcctttcctaataaaatgaggaaattgcatcgcattgtctgagtaggtgtcattctattctgg   6880
tccacggtgagggtgacaggaaaggattattttactcctttaacgtagcgtaacagactcatccacagtaagataagacc
bGH poly(A) signal

ggggtggggtggggcaggacagcaagggggaggattgggaagagaatagcaggcatgctggggagcggccAACgtacgca   6960
ccccaccccaccccgtcctgtcgttccccctcctaacccttctcttatcgtccgtacgacccctcgccggTTGcatgcgt
bGH poly(A) signal

GCggccGC   3'
              6968
CGccggCG   5'
```

Supplementary Figure S3. DESKGEN on-target and off-target analysis of *Tnfsf11*-targeted sgRNAs.

Tnfsf11-sg1

Sequence	PAM	Exon	Activity	Off-target
GGAGGCCAGCTCTCTCCACG	AGG	P	73	14

Guide Details	
Cut site	chr14 [+78,308,081 : -78,308,081]
GC %	70
Activity	73
Off-target	14

OFF-TARGET ANALYSIS:

Mismatches	0	1	2	3	Total
Coding	0	0	0	0	0
Non-Coding	0	5	69	598	672

OFF-TARGET HITS:

Sequence	PAM	Similarity	Mism.	Gene	Locus
GGAGGCCAGCTCTCTCCAGG	TGG	32	19	No	chrX@ 108697291-108697314
GGAGGCCAGCTCTCTCCAGG	TGG	32	19	No	chrX@ 20108906-20108929
GGAGGCCAGCTCTCTCCAAG	TGG	32	19	No	chr18@ 39797402-39797425
GGAGGCCAGCTCTCTCCGCG	AGG	20	18	No	chr15@ 33061846-33061869
GGAGGCCAGCTCTCTCCCCG	TGG	20	18	No	chr9@ 9132446-9132469
GGAGACCAGCTCTCTCCACA	CAG	6	5,20	No	chr17@ 23435687-23435710
GGAGACCAGCTCTCTCCACT	CAG	6	5,20	No	chr17@ 23362771-23362794
GGAGTCCAGCTCTCTCCACA	CAG	6	5,20	No	chr9@ 62856165-62856188
GAAGGCCAGCTCTCTCCAGG	TGG	6	2,19	No	chrX@ 118850871-118850894
GGTGGCCAGCTCTCTCCAGG	AGG	5	3,19	No	chr15@ 61496638-61496661
GGGGGCCAGCTCTCTCCAGG	CAG	5	3,19	No	chr7@ 18083920-18083943
GGAGACCAGCTCTCTCCAGG	AAG	4	5,19	No	chrX@ 132668125-132668148
GGAGACCAGCTCTCTCCAGG	CAG	4	5,19	No	chrX@ 104331095-104331118
GGAGTCCAGCTCTCTCCAGG	TGG	4	5,19	No	chrX@ 115474805-115474828

Sequence	PAM	Similarity	Mism.	Gene	Locus
GGAGACCAGCTCTCTCCAGG	CAG	4	5,19	No	chrX@ 162436757-162436780
GGAGACCAGCTCTCTCCAGG	AGG	4	5,19	No	chrX@ 158080260-158080283
GGAGACCAGCTCTCTCCAGG	TGG	4	5,19	No	chrX@ 16246963-16246986
GGAGACCAGCTCTCTCCAGG	TGG	4	5,19	No	chrX@ 16111349-16111372
GGAGACCAGCTCTCTCCAGG	CAG	4	5,19	No	chr18@ 17560826-17560849
GGAGACCAGCTCTCTCCAGG	CAG	4	5,19	No	chr18@ 15374510-15374533
GGAGACCAGCTCTCTCCAGG	AGG	4	5,19	No	chr17@ 81258623-81258646
GGAGACCAGCTCTCTCCAGG	AGG	4	5,19	No	chr17@ 53050125-53050148
GGAGACCAGCTCTCTCCAGG	CGG	4	5,19	No	chr16@ 56141624-56141647
GGAGACCAGCTCTCTCCAGG	CGG	4	5,19	No	chr16@ 35876138-35876161
GGAGACCAGCTCTCTCCAGG	CGG	4	5,19	No	chr14@ 112135213-112135236
GGAGACCAGCTCTCTCCAGG	AAG	4	5,19	No	chr14@ 8829035-8829058
GGAGACCAGCTCTCTCCAGG	TGG	4	5,19	No	chr12@ 109128063-109128086
GGAGACCAGCTCTCTCCAGG	AAG	4	5,19	No	chr12@ 45247102-45247125
GGAGACCAGCTCTCTCCAGG	TGG	4	5,19	No	chr10@ 125469397-125469420
GGAGACCAGCTCTCTCCAGG	CAG	4	5,19	No	chr10@ 17187475-17187498
GGAGACCAGCTCTCTCCAGG	CAG	4	5,19	No	chr10@ 78766165-78766188
GGAGACCAGCTCTCTCCAGG	TAG	4	5,19	No	chr10@ 57550598-57550621
GGAGACCAGCTCTCTCCAGG	TGG	4	5,19	No	chr9@ 37973121-37973144
GGAGACCAGCTCTCTCCAGG	TGG	4	5,19	No	chr8@ 59557762-59557785
GGAGACCAGCTCTCTCCAGG	TGG	4	5,19	No	chr8@ 73742415-73742438
GGAGACCAGCTCTCTCCAGG	CAG	4	5,19	No	chr7@ 129906529-129906552
GGAGACCAGCTCTCTCCAGG	CAG	4	5,19	No	chr7@ 65184338-65184361
GGAGACCAGCTCTCTCCAGG	AGG	4	5,19	No	chr7@ 58282921-58282944
GGAGACCAGCTCTCTCCAGG	TGG	4	5,19	No	chr6@ 63225175-63225198
GGAGACCAGCTCTCTCCAGG	CAG	4	5,19	No	chr6@ 129766229-129766252
GGAGACCAGCTCTCTCCAGG	CAG	4	5,19	No	chr6@ 44688564-44688587
GGAGACCAGCTCTCTCCAGG	CAG	4	5,19	No	chr5@ 126675997-126676020
GGAGACCAGCTCTCTCCAGG	AAG	4	5,19	No	chr5@ 87359227-87359250
GGAGACCAGCTCTCTCCAGG	AAG	4	5,19	No	chr5@ 87300749-87300772
GGAGACCAGCTCTCTCCAGG	TGG	4	5,19	No	chr4@ 73184232-73184255
GGAGACCAGCTCTCTCCAGG	CAG	4	5,19	No	chr3@ 56335965-56335988
GGAGACCAGCTCTCTCCAGG	TGG	4	5,19	No	chr3@ 17915223-17915246
GGAGACCAGCTCTCTCCAGG	AGG	4	5,19	No	chr3@ 58954535-58954558
GGAGACCAGCTCTCTCCAGG	TGG	4	5,19	No	chr3@ 21256459-21256482
GGAGACCAGCTCTCTCCAGG	CAG	4	5,19	No	chr3@ 10502946-10502969
GGAGACCAGCTCTCTCCAGG	CAG	4	5,19	No	chr2@ 14357004-14357027
GGAGACCAGCTCTCTCCAGG	CGG	4	5,19	No	chr2@ 139465469-139465492
GGAGACCAGCTCTCTCCAGG	AAG	4	5,19	No	chr2@ 97696308-97696331
GGAGACCAGCTCTCTCCAGG	AGG	4	5,19	No	chr2@ 78767220-78767243
GGAGACCAGCTCTCTCCAGG	TGG	4	5,19	No	chr2@ 40385442-40385465
GGAGACCAGCTCTCTCCAGG	AGG	4	5,19	No	chr2@ 3549485-3549508
GGAGACCAGCTCTCTCCAGG	TGG	4	5,19	No	chr1@ 99078626-99078649

Sequence	PAM	Similarity	Mism.	Gene	Locus
GGAGACCAGCTCTCTCCAGG	AGG	4	5,19	No	chr1@ 97535487-97535510
GGAGACCAGCTCTCTCCAGG	AGG	4	5,19	No	chr1@ 153344611-153344634
GGAGACCAGCTCTCTCCAGG	CAG	4	5,19	No	chr1@ 12558470-12558493
GCCGGCCAGGTCTCTCCACG	GAG	2	2,3,10	No	chr4@ 149307232-149307255
TGCAGCCAGCTCTCTCCACG	AGG	2	1,3,4	No	chr3@ 122455163-122455186
GGAGGCCAGTTCTCTCCAGG	AGG	2	10,19	No	chrX@ 20978967-20978990
GGAGGCCAGTTCTCTCCAGG	AGG	2	10,19	No	chrX@ 117739433-117739456
GGAGACCAGCTCTCTCCGCG	CAG	2	5,18	No	chr18@ 21487921-21487944
GGAGACCAGCTCTCTCCTCG	TAG	2	5,18	No	chr9@ 6834090-6834113
GGAGACCAGCTCTCTCCTCG	GGG	2	5,18	No	chr3@ 115174342-115174365
GGTGTCCATCTCTCTCCACG	TGG	2	3,5,9	No	chr3@ 32720611-32720634
AGAAGTCAGCTCTCTCCACG	GAG	2	1,4,6	No	chr2@ 162607400-162607423
GGTGGCCAGATCTCTCCACA	GAG	1	3,10,20	No	chr1@ 75651902-75651925
GCAGGCCAGGTTTCTCCACG	GGG	1	2,10,12	No	chr10@ 74973355-74973378
TGAGACCAGCTCTCTCCAGG	CAG	1	1,5,19	No	chrX@ 168984830-168984853
AGAGACCAGCTCTCTCCAGG	CAG	1	1,5,19	No	chr9@ 9367591-9367614
CGAGACCAGCTCTCTCCAAG	TAG	1	1,5,19	No	chr9@ 36156455-36156478
AGAGACCAGCTCTCTCCAGG	TGG	1	1,5,19	No	chr5@ 86223214-86223237
AGAGACCAGCTCTCTCCAGG	AGG	1	1,5,19	No	chr5@ 39209800-39209823
TGAGCCCAGCTCTCTCCAGG	TGG	1	1,5,19	No	chr3@ 128026647-128026670
GGAGGCCAGCTGTCTCCAGG	CAG	1	12,19	No	chr10@ 30324091-30324114
GGAGGCCAGCTTTCTCCAAG	TGG	1	12,19	No	chr3@ 16784007-16784030
GAAGACCAGCTCTCTCCAGG	GAG	1	2,5,19	No	chr8@ 62433436-62433459
GAAGACCAGCTCTCTCCAGG	TAG	1	2,5,19	No	chr8@ 65240531-65240554
GGTGACCAGCTCTCTCCAAG	CGG	1	3,5,19	No	chr4@ 21595857-21595880
GGTGACCAGCTCTCTCCAGG	TGG	1	3,5,19	No	chr2@ 55899886-55899909
GGAAACCAGCTCTCTCCAAG	TAG	1	4,5,19	No	chr19@ 19374333-19374356
GGAAACCAGCTCTCTCCAGG	CAG	1	4,5,19	No	chr17@ 53050344-53050367
GGAAACCAGCTCTCTCCAGG	AGG	1	4,5,19	No	chr16@ 45505898-45505921
GGAAACCAGCTCTCTCCAGG	CAG	1	4,5,19	No	chr15@ 17733445-17733468
GGAAACCAGCTCTCTCCAGG	CAG	1	4,5,19	No	chr12@ 4324463-4324486
GGAGACCCGCTCTCTCCAGG	TGG	1	5,8,19	No	chrX@ 140785952-140785975
GGAGACCTGCTCTCTCCAGG	CAG	1	5,8,19	No	chr18@ 31098351-31098374
GGAGACCTGCTCTCTCCAGG	AGG	1	5,8,19	No	chr17@ 54034089-54034112
GGAGACCTGCTCTCTCCAGG	CAG	1	5,8,19	No	chr16@ 60071452-60071475
GGAGACCTGCTCTCTCCAGG	TGG	1	5,8,19	No	chr14@ 36417759-36417782
GGAGACCTGCTCTCTCCATG	CAG	1	5,8,19	No	chr3@ 44087956-44087979
GGAGGCAAGCTCTCTGCACG	TGG	1	7,16	No	chr15@ 100587086-100587109
AGAGGCCAACTCTCTCCACT	GAG	1	1,9,20	No	chr3@ 52257491-52257514
GGAGACTAGCTCTCTCCACT	GAG	1	5,7,20	No	chr7@ 62494843-62494866
GGAGACTAGCTCTCTCCACT	GAG	1	5,7,20	No	chr4@ 25357948-25357971
GGAGACTAGCTCTCTCCACT	GAG	1	5,7,20	No	chr3@ 4114639-4114662
GGAGACCAGTTCTCTCCAGG	AGG	1	5,10,19	No	chrX@ 89780376-89780399

Sequence	PAM	Similarity	Mism.	Gene	Locus
GGAGACCAGTTCTCTCCAGG	AAG	1	5,10,19	No	chrX@ 46929236-46929259
GGAGACCAGTTCTCTCCAGG	AGG	1	5,10,19	No	chrX@ 109208577-109208600
GGAGACCAGTTCTCTCCAGG	AAG	1	5,10,19	No	chrX@ 115820033-115820056
GGAGACCAGTTCTCTCCAAG	AGG	1	5,10,19	No	chrX@ 137220364-137220387
GGAGACCAGTTCTCTCCAGG	TGG	1	5,10,19	No	chr19@ 9795872-9795895
GGAGACCAGGTCTCTCCAGG	TGG	1	5,10,19	No	chr18@ 16849295-16849318
GGAGACCAGTTCTCTCCAGG	AGG	1	5,10,19	No	chr18@ 58746595-58746618
GGAGACCAGTTCTCTCCAGG	AGG	1	5,10,19	No	chr18@ 33126397-33126420
GGAGACCAGTTCTCTCCAGG	AGG	1	5,10,19	No	chr18@ 37470174-37470197
GGAGACCAGTTCTCTCCAGG	AGG	1	5,10,19	No	chr18@ 81780362-81780385
GGAGACCAGTTCTCTCCATG	TGG	1	5,10,19	No	chr18@ 44163004-44163027
GGAGACCAGTTCTCTCCATG	AGG	1	5,10,19	No	chr17@ 42680247-42680270
GGAGACCAGTTCTCTCCATG	AGG	1	5,10,19	No	chr16@ 14562339-14562362
GGAGACCAGTTCTCTCCAGG	AGG	1	5,10,19	No	chr16@ 65127167-65127190
GGAGACCAGTTCTCTCCAGG	AGG	1	5,10,19	No	chr16@ 90638819-90638842
GGAGACCAGTTCTCTCCAGG	AGG	1	5,10,19	No	chr16@ 14429112-14429135
GGAGACCAGTTCTCTCCAGG	AAG	1	5,10,19	No	chr16@ 47348780-47348803
GGAGACCAGTTCTCTCCAGG	AGG	1	5,10,19	No	chr16@ 44023929-44023952
GGAGACCAGGTCTCTCCAGG	CGG	1	5,10,19	No	chr16@ 15713440-15713463
GGAGACCAGTTCTCTCCAGG	AGG	1	5,10,19	No	chr16@ 9179674-9179697
GGAGACCAGTTCTCTCCAGG	AGG	1	5,10,19	No	chr16@ 37341680-37341703
GGAGACCAGTTCTCTCCAGG	AGG	1	5,10,19	No	chr14@ 38243427-38243450
GGAGACCAGATCTCTCCAGG	CAG	1	5,10,19	No	chr14@ 13713691-13713714
GGAGACCAGTTCTCTCCATG	AGG	1	5,10,19	No	chr13@ 82547471-82547494
GGAGACCAGTTCTCTCCAGG	AGG	1	5,10,19	No	chr13@ 11252799-11252822
GGAGACCAGTTCTCTCCAGG	AGG	1	5,10,19	No	chr12@ 29504143-29504166
GGAGACCAGTTCTCTCCAGG	AGG	1	5,10,19	No	chr12@ 70463955-70463978
GGAGACCAGTTCTCTCCAGG	AAG	1	5,10,19	No	chr12@ 78629466-78629489
GGAGACCAGTTCTCTCCAGG	AAG	1	5,10,19	No	chr12@ 97573977-97574000
GGAGACCAGTTCTCTCCAGG	AGG	1	5,10,19	No	chr12@ 70381198-70381221
GGAGACCAGTTCTCTCCAGG	TGG	1	5,10,19	No	chr12@ 28264762-28264785
GGAGACCAGTTCTCTCCAGG	AGG	1	5,10,19	No	chr11@ 29437206-29437229
GGAGACCAGTTCTCTCCATG	AGG	1	5,10,19	No	chr11@ 18659856-18659879
GGAGACCAGTTCTCTCCAGG	AGG	1	5,10,19	No	chr11@ 40935581-40935604
GGAGACCAGGTCTCTCCATG	AAG	1	5,10,19	No	chr11@ 14954692-14954715
GGAGACCAGTTCTCTCCAGG	AGG	1	5,10,19	No	chr10@ 8163467-8163490
GGAGACCAGTTCTCTCCAGG	AGG	1	5,10,19	No	chr10@ 109235117-109235140
GGAGACCAGTTCTCTCCAGG	TGG	1	5,10,19	No	chr10@ 17187678-17187701
GGAGACCAGTTCTCTCCAGG	AGG	1	5,10,19	No	chr9@ 33737079-33737102
GGAGACCAGTTCTCTCCAGG	AGG	1	5,10,19	No	chr9@ 3989472-3989495
GGAGACCAGTTCTCTCCAGG	AGG	1	5,10,19	No	chr9@ 90468558-90468581
GGAGACCAGTTCTCTCCAGG	AGG	1	5,10,19	No	chr9@ 71987228-71987251
GGAGACCAGTTCTCTCCAGG	AGG	1	5,10,19	No	chr9@ 66341383-66341406

Sequence	PAM	Similarity	Mism.	Gene	Locus
GGAGACCAGTTCTCTCCAGG	AGG	1	5,10,19	No	chr8@ 90748768-90748791
GGAGACCAGTTCTCTCCAGG	AGG	1	5,10,19	No	chr8@ 60876292-60876315
GGAGACCAGTTCTCTCCAGG	AGG	1	5,10,19	No	chr7@ 5281841-5281864
GGAGACCAGTTCTCTCCAGG	AGG	1	5,10,19	No	chr7@ 53761800-53761823
GGAGACCAGTTCTCTCCAGG	AGG	1	5,10,19	No	chr6@ 106934863-106934886
GGAGACCAGTTCTCTCCAGG	CAG	1	5,10,19	No	chr6@ 122260887-122260910
GGAGACCAGTTCTCTCCAGG	AGG	1	5,10,19	No	chr6@ 24805249-24805272
GGAGACCAGTTCTCTCCAGG	AGG	1	5,10,19	No	chr6@ 128679312-128679335
GGAGACCAGTTCTCTCCAAG	TGG	1	5,10,19	No	chr5@ 7094456-7094479
GGAGACCAGTTCTCTCCAGG	AGG	1	5,10,19	No	chr5@ 34683225-34683248
GGAGACCAGTTCTCTCCAGG	AGG	1	5,10,19	No	chr4@ 61550975-61550998
GGAGACCAGTTCTCTCCAGG	AGG	1	5,10,19	No	chr4@ 60694051-60694074
GGAGACCAGTTCTCTCCAGG	AGG	1	5,10,19	No	chr4@ 61468936-61468959
GGAGACCAGTTCTCTCCAGG	AGG	1	5,10,19	No	chr4@ 61179393-61179416
GGAGACCAGTTCTCTCCAGG	AGG	1	5,10,19	No	chr4@ 60460594-60460617
GGAGACCAGTTCTCTCCAGG	AGG	1	5,10,19	No	chr4@ 60533326-60533349
GGAGACCAGTTCTCTCCAGG	AGG	1	5,10,19	No	chr4@ 60772707-60772730
GGAGACCAGTTCTCTCCAGG	AGG	1	5,10,19	No	chr4@ 39751087-39751110
GGAGACCAGTTCTCTCCAGG	AGG	1	5,10,19	No	chr4@ 55836534-55836557
GGAGACCAGTTCTCTCCAGG	AGG	1	5,10,19	No	chr4@ 132092626-132092649
GGAGACCAGTTCTCTCCAGG	AGG	1	5,10,19	No	chr4@ 90521707-90521730
GGAGACCAGTTCTCTCCAGG	AGG	1	5,10,19	No	chr4@ 61624987-61625010
GGAGACCAGATCTCTCCAGG	AGG	1	5,10,19	No	chr4@ 80022302-80022325
GGAGACCAGTTCTCTCCAGG	AAG	1	5,10,19	No	chr4@ 60806480-60806503
GGAGACCAGTTCTCTCCAGG	AAG	1	5,10,19	No	chr4@ 61658973-61658996
GGAGACCAGTTCTCTCCAGG	AAG	1	5,10,19	No	chr4@ 60728036-60728059
GGAGACCAGTTCTCTCCAGG	AAG	1	5,10,19	No	chr4@ 61504399-61504422
GGAGACCAGTTCTCTCCAGG	AAG	1	5,10,19	No	chr4@ 60124760-60124783
GGAGACCAGTTCTCTCCAGG	AAG	1	5,10,19	No	chr4@ 61580767-61580790
GGAGACCAGTTCTCTCCAGG	AAG	1	5,10,19	No	chr4@ 61293306-61293329
GGAGACCAGTTCTCTCCAGG	AAG	1	5,10,19	No	chr4@ 60399391-60399414
GGAGACCAGTTCTCTCCAGG	AGG	1	5,10,19	No	chr4@ 53366849-53366872
GGAGACCAGTTCTCTCCAGG	CAG	1	5,10,19	No	chr4@ 6099047-6099070
GGAGACCAGTTCTCTCCAGG	AGG	1	5,10,19	No	chr4@ 105521567-105521590
GGAGACCAGTTCTCTCCAGG	TAG	1	5,10,19	No	chr4@ 12587583-12587606
GGAGACCAGTTCTCTCCAGG	AAG	1	5,10,19	No	chr4@ 60207444-60207467
GGAGACCAGTTCTCTCCAGG	AAG	1	5,10,19	No	chr4@ 60647314-60647337
GGAGACCAGTTCTCTCCAGG	AAG	1	5,10,19	No	chr4@ 61759603-61759626
GGAGACCAGTTCTCTCCAGG	AAG	1	5,10,19	No	chr4@ 60567099-60567122
GGAGACCAGTTCTCTCCAGG	AAG	1	5,10,19	No	chr4@ 61213163-61213186
GGAGACCAGATCTCTCCATG	AGG	1	5,10,19	No	chr3@ 134151759-134151782
GGAGACCAGATCTCTCCAGG	CAG	1	5,10,19	No	chr3@ 4811857-4811880
GGAGACCAGTTCTCTCCAGG	AGG	1	5,10,19	No	chr3@ 126175718-126175741

Sequence	PAM	Similarity	Mism.	Gene	Locus
GGAGACCAGTTCTCTCCAGG	AGG	1	5,10,19	No	chr3@ 36305867-36305890
GGAGACCAGTTCTCTCCAGG	AAG	1	5,10,19	No	chr3@ 53782761-53782784
GGAGACCAGTTCTCTCCAGG	AGG	1	5,10,19	No	chr3@ 83824391-83824414
GGAGACCAGTTCTCTCCAGG	AGG	1	5,10,19	No	chr3@ 26868466-26868489
GGAGACCAGTTCTCTCCATG	AGG	1	5,10,19	No	chr3@ 37100399-37100422
GGAGACCAGGTCTCTCCAAG	AGG	1	5,10,19	No	chr2@ 47779375-47779398
GGAGACCAGTTCTCTCCAGG	AGG	1	5,10,19	No	chr2@ 23934305-23934328
GGAGACCAGTTCTCTCCAGG	AGG	1	5,10,19	No	chr2@ 154976368-154976391
GGAGACCAGTTCTCTCCATG	AGG	1	5,10,19	No	chr1@ 9347505-9347528
GGAGACCAGTTCTCTCCAGG	AGG	1	5,10,19	No	chr1@ 22991775-22991798
GGAGACCAGTTCTCTCCAGG	AAG	1	5,10,19	No	chr1@ 117799970-117799993
GGAGACCAGTTCTCTCCAGG	AGG	1	5,10,19	No	chr1@ 43728454-43728477
GGAGACCAGTTCTCTCCAGG	AGG	1	5,10,19	No	chr1@ 97428908-97428931
GGAGACCAGTTCTCTCCAGG	AGG	1	5,10,19	No	chr1@ 60965911-60965934
GGAGACCAGTTCTCTCCAGG	AGG	1	5,10,19	No	chr1@ 30460014-30460037
GGAGACCAGTTCTCTCCAGG	AGG	1	5,10,19	No	chr1@ 58010917-58010940
GGAGACCAGTTCTCTCCAGG	AGG	1	5,10,19	No	chr1@ 138313954-138313977
GGAGACCAGTTCTCTCCAGG	AAG	1	5,10,19	No	chr1@ 150879970-150879993
GGAGACCAGTTCTCTCCAGG	AGG	1	5,10,19	No	chr1@ 122170509-122170532
GGAGACCAGTTCTCTCCATG	AAG	1	5,10,19	No	chr1@ 5765312-5765335
GGAGCACAGCTCTCTCCACT	TAG	1	5,6,20	No	chr11@ 23851387-23851410
GTAGGCAAGCTCTCTCCAAG	TAG	1	2,7,19	No	chr9@ 110441825-110441848
GGAAGCCAGCCTTCTCCACG	TAG	1	4,11,12	No	chr3@ 9326204-9326227
CGAGACCAGCTCTCTCCTCG	CAG	1	1,5,18	No	chr4@ 75164426-75164449
GGAGACTAGCTCTCTCCAGG	CGG	1	5,7,19	No	chr17@ 80591622-80591645
GGAGACAAGCTCTCTCCAGG	AAG	1	5,7,19	No	chr17@ 38411586-38411609
GGAGACTAGCTCTCTCCAGG	CAG	1	5,7,19	No	chr13@ 83962172-83962195
GGAGACTAGCTCTCTCCAGG	CAG	1	5,7,19	No	chr12@ 74123885-74123908
GGAGACTAGCTCTCTCCAGG	TGG	1	5,7,19	No	chr12@ 65162500-65162523
GGAGACTAGCTCTCTCCAGG	CAG	1	5,7,19	No	chr10@ 53135537-53135560
GGAGACTAGCTCTCTCCAGG	CAG	1	5,7,19	No	chr6@ 14544131-14544154
GGAGACTAGCTCTCTCCAGG	TGG	1	5,7,19	No	chr1@ 3189390-3189413
GAAGACCAGCTCTCTCCCCG	CAG	1	2,5,18	No	chr7@ 12861574-12861597
GGAGACCAACTCTCTCCAAG	AGG	1	5,9,19	No	chr19@ 53593106-53593129
GGAGACCATCTCTCTCCAAG	TGG	1	5,9,19	No	chr7@ 13105394-13105417
GGAGATCAGCTCTCTCCATG	TGG	1	5,6,19	No	chr17@ 22812194-22812217
GGAGAACAGCTCTCTCCAGG	CAG	1	5,6,19	No	chr13@ 81212528-81212551
GGAGATCAGCTCTCTCCAGG	CAG	1	5,6,19	No	chr13@ 16533168-16533191
GGAGAGCAGCTCTCTCCAAG	TGG	1	5,6,19	No	chr11@ 18100814-18100837
GGAGATCAGCTCTCTCCAAG	CAG	1	5,6,19	No	chr10@ 19058379-19058402
GGAGGCAAGGTCTCTCCATG	TAG	1	7,10,19	No	chr18@ 77053921-77053944
GGAGACCAGTTCTCTCCTCG	AGG	1	5,10,18	No	chr1@ 110093686-110093709
GGAGACCAGCACTCTCCAGG	TGG	1	5,11,19	No	chrX@ 14077860-14077883

Sequence	PAM	Similarity	Mism.	Gene	Locus
GGAGACCAGCCCTCTCCAGG	CAG	1	5,11,19	No	chr17@ 57512043-57512066
GGAGACCAGCCCTCTCCAAG	AGG	1	5,11,19	No	chr3@ 20766670-20766693
GGCTGCCAGCTCTCTTCACG	GAG	1	3,4,16	No	chr4@ 62454367-62454390
GGAGACCAGCTGTCTCCAGG	AAG	0	5,12,19	No	chr19@ 11437379-11437402
GGAGACCAGCTTTCTCCAGG	CAG	0	5,12,19	No	chr15@ 5626736-5626759
GGAGACCAGCTTTCTCCAGG	TGG	0	5,12,19	No	chr12@ 33529852-33529875
GGAGACCAGCTGTCTCCAGG	CAG	0	5,12,19	No	chr12@ 74960697-74960720
GGAGACCAGCTTTCTCCAGG	TGG	0	5,12,19	No	chr11@ 99975672-99975695
GGAGACCAGCTGTCTCCATG	TGG	0	5,12,19	No	chr4@ 12209796-12209819
GGAGACCAGCTATCTCCAGG	GGG	0	5,12,19	No	chr4@ 17397847-17397870
GGAGACCAGCTGTCTCCAGG	TGG	0	5,12,19	No	chr3@ 9386462-9386485
GGAGACCAGCTCTCTCCAGA	AGG	0	5,19,20	No	chr15@ 48874169-48874192
GGAGACCAGCTCTCTCCAGA	CAG	0	5,19,20	No	chr13@ 79852067-79852090
GGAGACCAGCTCTCTCCAGT	AGG	0	5,19,20	No	chr13@ 84143776-84143799
GGAGACCAGCTCTCTCCATC	AGG	0	5,19,20	No	chr12@ 68842098-68842121
GGAGACCAGCTCTCTCCAGA	CAG	0	5,19,20	No	chr11@ 19125885-19125908
GGAGACCAGCTCTCTCCAGA	CAG	0	5,19,20	No	chr9@ 36454737-36454760
GGAGACCAGCTCTCTCCAGA	TAG	0	5,19,20	No	chr2@ 49230167-49230190
GGAGGCCAGTTTTCTCCAGG	AGG	0	10,12,19	No	chr9@ 8532588-8532611
GGAGGCCAGTTTTCTCCAGG	AGG	0	10,12,19	No	chr2@ 23420508-23420531
GGAGACCAGCTCTCTCTAGG	CAG	0	5,17,19	No	chrX@ 160751952-160751975
GGAGACCAGCTCTCTCTAGG	CAG	0	5,17,19	No	chrX@ 158080467-158080490
GGAGACCAGCTCTCTCTAAG	CAG	0	5,17,19	No	chr17@ 54865520-54865543
GGAGACCAGCTCTCTCAAGG	TGG	0	5,17,19	No	chr3@ 72132813-72132836
GGAGACCAGCTCTCTCTAGG	TAG	0	5,17,19	No	chr2@ 39633461-39633484
GGAGACCAGCTCTCTCTAGG	TGG	0	5,17,19	No	chr1@ 113834491-113834514
GGAGACCAGCTCTCTCTAGG	CAG	0	5,17,19	No	chr1@ 99175859-99175882
GGAGTCCAACTCTCTCCTCG	TGG	0	5,9,18	No	chr17@ 91345646-91345669
GGAGGCCAGCTCTCTCCTTG	CAG	0	18,19	No	chr13@ 14917332-14917355
GGAGGCCAGCTCTCTCCTGG	TGG	0	18,19	No	chr10@ 31368600-31368623
GGAGGCCAGCTCTCTCCCAG	CAG	0	18,19	No	chr5@ 26713413-26713436
GGAGGCCAGCTCTCTCCTGG	TGG	0	18,19	No	chr2@ 55374134-55374157
GGAGGCCAGCTCTCTCCTGG	TGG	0	18,19	No	chr2@ 61433597-61433620
GGAGGCCAGCTCTCTCCCAG	TGG	0	18,19	No	chr1@ 5630490-5630513
GGAGGCCAGTTCTCTCTAGG	AGG	0	10,17,19	No	chr1@ 121985410-121985433
GGAGACCAGCTCTCCCCAGG	AAG	0	5,15,19	No	chr11@ 10737105-10737128
GGAGACCAGCTCTCCCCAAG	CAG	0	5,15,19	No	chr9@ 3667331-3667354
GGAGACCAGCTCTCTCCTCT	GAG	0	5,18,20	No	chrX@ 40027631-40027654
GGAGACCAGCTCTCTCCTCT	GAG	0	5,18,20	No	chrX@ 24373068-24373091
GGAGACCAGCTCTCTCCTCA	TAG	0	5,18,20	No	chr18@ 22076380-22076403
GGAGACCAGCTCTCTCCTCT	GAG	0	5,18,20	No	chr17@ 41676952-41676975
GGAGACCAGCTCTCTCCCCA	GGG	0	5,18,20	No	chr16@ 20186114-20186137
GGAGACCAGCTCTCTCCTCT	GAG	0	5,18,20	No	chr15@ 57292172-57292195

Sequence	PAM	Similarity	Mism.	Gene	Locus
GGAGACCAGCTCTCTCCTCT	GAG	0	5,18,20	No	chr14@ 52503564-52503587
GGAGACCAGCTCTCTCCTCT	GAG	0	5,18,20	No	chr7@ 68625448-68625471
GGAGACCAGCTCTCTCCTCT	GAG	0	5,18,20	No	chr6@ 61482264-61482287
GGAGACCAGCTCTCTCCTCT	GAG	0	5,18,20	No	chr5@ 14552258-14552281
GGAGACCAGCTCTCTCCTCA	TGG	0	5,18,20	No	chr5@ 5919297-5919320
GGAGACCAGCTCTCTCCTCT	GAG	0	5,18,20	No	chr4@ 29359308-29359331
GGAGACCAGCTCTCTCCTCT	GAG	0	5,18,20	No	chr4@ 13680420-13680443
GGAGACCAGCTCTCTCCTCT	GAG	0	5,18,20	No	chr4@ 4170753-4170776
GGAGACCAGCTCTCTCCTCT	GAG	0	5,18,20	No	chr3@ 41786597-41786620
GGAGACCAGCTCTCTCCTCT	GAG	0	5,18,20	No	chr2@ 10952649-10952672
GGAGACCAGCTCTCTCCTCT	GAG	0	5,18,20	No	chr2@ 5275979-5276002
GGAGACCAGCTCTCTCCTCT	GAG	0	5,18,20	No	chr1@ 107233146-107233169
GGAGGCTAGCTGTCACCACG	GGG	0	7,12,15	No	chr19@ 32726447-32726470
GGAGACCAGCTCACTCCCCG	TGG	0	5,13,18	No	chr16@ 15921746-15921769
AGAGGCCAGCTCTCTCCTGG	TGG	0	1,18,19	No	chrX@ 38840120-38840143
TGAGGCCAGCTCTCTCCTAG	CAG	0	1,18,19	No	chr14@ 107171340-107171363
GGAAGCCAGCTCTCTCCTGG	CAG	0	4,18,19	No	chrX@ 110974290-110974313
GGAAGCCAGCTCTCTCCCAG	TGG	0	4,18,19	No	chr18@ 39169211-39169234
GGAAGCCAGCTCTCTCCTGG	CAG	0	4,18,19	No	chr17@ 43290143-43290166
GGAGGCCAGCCCTCTCCAGA	GGG	0	11,19,20	No	chr17@ 84860919-84860942
GGAGGCCAGCACTCTCCAAA	CAG	0	11,19,20	No	chr17@ 52668262-52668285
GGAGACCAGCTCTCTCCCAG	CAG	0	5,18,19	No	chrX@ 104515425-104515448
GGAGACCAGCTCTCTCCCAG	TGG	0	5,18,19	No	chrX@ 103048235-103048258
GGAGACCAGCTCTCTCCCAG	TGG	0	5,18,19	No	chrX@ 71508862-71508885
GGAGACCAGCTCTCTCCTGG	TGG	0	5,18,19	No	chrX@ 23627853-23627876
GGAGACCAGCTCTCTCCTGG	TAG	0	5,18,19	No	chrX@ 74587633-74587656
GGAGACCAGCTCTCTCCTGG	CAG	0	5,18,19	No	chrX@ 144056104-144056127
GGAGACCAGCTCTCTCCTGG	TAG	0	5,18,19	No	chrX@ 14091184-14091207
GGAGACCAGCTCTCTCCTGG	TAG	0	5,18,19	No	chrX@ 38839937-38839960
GGAGACCAGCTCTCTCCTGG	TAG	0	5,18,19	No	chrX@ 91073603-91073626
GGAGACCAGCTCTCTCCCAG	CAG	0	5,18,19	No	chrX@ 9084458-9084481
GGAGACCAGCTCTCTCCCAG	CAG	0	5,18,19	No	chrX@ 9188246-9188269
GGAGACCAGCTCTCTCCTGG	TGG	0	5,18,19	No	chrX@ 21040643-21040666
GGAGACCAGCTCTCTCCTGG	AAG	0	5,18,19	No	chrX@ 59995959-59995982
GGAGACCAGCTCTCTCCTGG	CAG	0	5,18,19	No	chrX@ 80309561-80309584
GGAGACCAGCTCTCTCCTGG	CAG	0	5,18,19	No	chrX@ 163972341-163972364
GGAGACCAGCTCTCTCCTGG	CAG	0	5,18,19	No	chrX@ 14034755-14034778
GGAGACCAGCTCTCTCCCAG	TGG	0	5,18,19	No	chr19@ 9796080-9796103
GGAGACCAGCTCTCTCCCAG	TGG	0	5,18,19	No	chr19@ 16788887-16788910
GGAGACCAGCTCTCTCCCAG	CAG	0	5,18,19	No	chr19@ 39077722-39077745
GGAGACCAGCTCTCTCCTGG	AAG	0	5,18,19	No	chr19@ 43008509-43008532
GGAGACCAGCTCTCTCCTGG	TGG	0	5,18,19	No	chr19@ 54985811-54985834
GGAGACCAGCTCTCTCCTGG	CAG	0	5,18,19	No	chr19@ 26329904-26329927

Sequence	PAM	Similarity	Mism.	Gene	Locus
GGAGACCAGCTCTCTCCTGG	CAG	0	5,18,19	No	chr19@ 38210039-38210062
GGAGACCAGCTCTCTCCTGG	TGG	0	5,18,19	No	chr19@ 11677996-11678019
GGAGACCAGCTCTCTCCCGG	AGG	0	5,18,19	No	chr18@ 14959871-14959894
GGAGACCAGCTCTCTCCCTG	TGG	0	5,18,19	No	chr18@ 14454726-14454749
GGAGACCAGCTCTCTCCTAG	CAG	0	5,18,19	No	chr18@ 33421439-33421462
GGAGACCAGCTCTCTCCTGG	CAG	0	5,18,19	No	chr18@ 9757563-9757586
GGAGACCAGCTCTCTCCTGG	TGG	0	5,18,19	No	chr18@ 47894092-47894115
GGAGACCAGCTCTCTCCTGG	TGG	0	5,18,19	No	chr18@ 79613577-79613600
GGAGACCAGCTCTCTCCTGG	CGG	0	5,18,19	No	chr18@ 31366516-31366539
GGAGACCAGCTCTCTCCTGG	TGG	0	5,18,19	No	chr18@ 52080842-52080865
GGAGACCAGCTCTCTCCCGG	TGG	0	5,18,19	No	chr18@ 22074653-22074676
GGAGACCAGCTCTCTCCTGG	CAG	0	5,18,19	No	chr18@ 32751081-32751104
GGAGACCAGCTCTCTCCTAG	TAG	0	5,18,19	No	chr18@ 29816557-29816580
GGAGTCCAGCTCTCTCCTGG	AAG	0	5,18,19	No	chr17@ 40547764-40547787
GGAGACCAGCTCTCTCCGGG	CAG	0	5,18,19	No	chr17@ 58791251-58791274
GGAGACCAGCTCTCTCCTGG	TGG	0	5,18,19	No	chr17@ 76094199-76094222
GGAGACCAGCTCTCTCCTGG	TGG	0	5,18,19	No	chr17@ 58077996-58078019
GGAGACCAGCTCTCTCCTGG	TGG	0	5,18,19	No	chr17@ 77530647-77530670
GGAGACCAGCTCTCTCCCAG	CAG	0	5,18,19	No	chr17@ 72728423-72728446
GGAGACCAGCTCTCTCCCGG	TGG	0	5,18,19	No	chr17@ 42680429-42680452
GGAGACCAGCTCTCTCCCTG	TGG	0	5,18,19	No	chr17@ 54154235-54154258
GGAGACCAGCTCTCTCCTGG	TGG	0	5,18,19	No	chr17@ 89102858-89102881
GGAGACCAGCTCTCTCCTGG	CAG	0	5,18,19	No	chr17@ 89585731-89585754
GGAGACCAGCTCTCTCCTGG	CAG	0	5,18,19	No	chr17@ 42969878-42969901
GGAGACCAGCTCTCTCCTGG	TGG	0	5,18,19	No	chr17@ 57465796-57465819
GGAGACCAGCTCTCTCCCAG	CGG	0	5,18,19	No	chr16@ 17545081-17545104
GGAGACCAGCTCTCTCCCGG	CAG	0	5,18,19	No	chr16@ 87624071-87624094
GGAGACCAGCTCTCTCCTGG	CAG	0	5,18,19	No	chr16@ 13166089-13166112
GGAGACCAGCTCTCTCCTGG	AAG	0	5,18,19	No	chr16@ 61676043-61676066
GGAGCCCAGCTCTCTCCCAG	CAG	0	5,18,19	No	chr16@ 36155002-36155025
GGAGTCCAGCTCTCTCCTGG	TGG	0	5,18,19	No	chr16@ 71751401-71751424
GGAGACCAGCTCTCTCCCAG	TGG	0	5,18,19	No	chr16@ 4245235-4245258
GGAGACCAGCTCTCTCCTGG	TGG	0	5,18,19	No	chr16@ 19709938-19709961
GGAGACCAGCTCTCTCCTGG	TGG	0	5,18,19	No	chr16@ 19709733-19709756
GGAGACCAGCTCTCTCCTGG	TGG	0	5,18,19	No	chr16@ 59858686-59858709
GGAGACCAGCTCTCTCCTGG	AGG	0	5,18,19	No	chr16@ 11830651-11830674
GGAGACCAGCTCTCTCCTGG	AGG	0	5,18,19	No	chr16@ 51317554-51317577
GGAGACCAGCTCTCTCCTGG	TGG	0	5,18,19	No	chr15@ 60358105-60358128
GGAGACCAGCTCTCTCCCAG	CAG	0	5,18,19	No	chr15@ 63179724-63179747
GGAGACCAGCTCTCTCCTGG	CAG	0	5,18,19	No	chr15@ 43239421-43239444
GGAGACCAGCTCTCTCCTGG	CGG	0	5,18,19	No	chr15@ 56899363-56899386
GGAGCCCAGCTCTCTCCTGG	CAG	0	5,18,19	No	chr14@ 13759070-13759093
GGAGACCAGCTCTCTCCCGG	CAG	0	5,18,19	No	chr14@ 105188615-105188638

Sequence	PAM	Similarity	Mism.	Gene	Locus
GGAGACCAGCTCTCTCCTGG	CAG	0	5,18,19	No	chr14@ 49274648-49274671
GGAGACCAGCTCTCTCCTGG	TGG	0	5,18,19	No	chr14@ 14946074-14946097
GGAGACCAGCTCTCTCCTGG	TGG	0	5,18,19	No	chr14@ 9012954-9012977
GGAGACCAGCTCTCTCCCGG	TGG	0	5,18,19	No	chr14@ 74433982-74434005
GGAGACCAGCTCTCTCCTGG	CAG	0	5,18,19	No	chr14@ 49056214-49056237
GGAGACCAGCTCTCTCCTGG	CAG	0	5,18,19	No	chr14@ 44998018-44998041
GGAGACCAGCTCTCTCCTGG	CAG	0	5,18,19	No	chr14@ 107872839-107872862
GGAGACCAGCTCTCTCCTGG	GAG	0	5,18,19	No	chr14@ 102769968-102769991
GGAGACCAGCTCTCTCCCAG	TGG	0	5,18,19	No	chr13@ 80762993-80763016
GGAGACCAGCTCTCTCCCTG	CAG	0	5,18,19	No	chr13@ 93210132-93210155
GGAGACCAGCTCTCTCCTAG	TGG	0	5,18,19	No	chr13@ 120060909-120060932
GGAGACCAGCTCTCTCCTGG	CAG	0	5,18,19	No	chr13@ 86521584-86521607
GGAGACCAGCTCTCTCCTGG	CAG	0	5,18,19	No	chr13@ 81033228-81033251
GGAGACCAGCTCTCTCCTGG	TGG	0	5,18,19	No	chr13@ 14339540-14339563
GGAGACCAGCTCTCTCCTGG	CAG	0	5,18,19	No	chr13@ 60281984-60282007
GGAGACCAGCTCTCTCCTGG	TGG	0	5,18,19	No	chr13@ 106329054-106329077
GGAGACCAGCTCTCTCCTGG	TGG	0	5,18,19	No	chr13@ 114414967-114414990
GGAGACCAGCTCTCTCCTGG	TGG	0	5,18,19	No	chr13@ 89484920-89484943
GGAGACCAGCTCTCTCCTGG	TGG	0	5,18,19	No	chr13@ 108590200-108590223
GGAGACCAGCTCTCTCCTGG	TGG	0	5,18,19	No	chr13@ 17265821-17265844
GGAGTCCAGCTCTCTCCTGG	TGG	0	5,18,19	No	chr13@ 84830187-84830210
GGAGACCAGCTCTCTCCCAG	TGG	0	5,18,19	No	chr13@ 9028250-9028273
GGAGACCAGCTCTCTCCCAG	TAG	0	5,18,19	No	chr13@ 85382609-85382632
GGAGACCAGCTCTCTCCTGG	CAG	0	5,18,19	No	chr13@ 91065115-91065138
GGAGACCAGCTCTCTCCTGG	TGG	0	5,18,19	No	chr13@ 86783473-86783496
GGAGACCAGCTCTCTCCTGG	CAG	0	5,18,19	No	chr13@ 21051031-21051054
GGAGACCAGCTCTCTCCTGG	CAG	0	5,18,19	No	chr13@ 94333638-94333661
GGAGACCAGCTCTCTCCTGG	CAG	0	5,18,19	No	chr13@ 4490893-4490916
GGAGACCAGCTCTCTCCTGG	TGG	0	5,18,19	No	chr13@ 92932642-92932665
GGAGACCAGCTCTCTCCTGG	TGG	0	5,18,19	No	chr13@ 14238828-14238851
GGAGACCAGCTCTCTCCTAG	TGG	0	5,18,19	No	chr13@ 120001597-120001620
GGAGACCAGCTCTCTCCCAG	TGG	0	5,18,19	No	chr12@ 60554402-60554425
GGAGACCAGCTCTCTCCCAG	CGG	0	5,18,19	No	chr12@ 87011167-87011190
GGAGACCAGCTCTCTCCCAG	CAG	0	5,18,19	No	chr12@ 68852804-68852827
GGAGACCAGCTCTCTCCTGG	CGG	0	5,18,19	No	chr12@ 112220163-112220186
GGAGACCAGCTCTCTCCTAG	TGG	0	5,18,19	No	chr12@ 109304260-109304283
GGAGACCAGCTCTCTCCTAG	TGG	0	5,18,19	No	chr12@ 6546967-6546990
GGAGACCAGCTCTCTCCTGG	CAG	0	5,18,19	No	chr12@ 49255319-49255342
GGAGACCAGCTCTCTCCTGG	TGG	0	5,18,19	No	chr12@ 48881604-48881627
GGAGACCAGCTCTCTCCCAG	CGG	0	5,18,19	No	chr12@ 81492539-81492562
GGAGACCAGCTCTCTCCCGG	TGG	0	5,18,19	No	chr12@ 110412122-110412145
GGAGACCAGCTCTCTCCTGG	TGG	0	5,18,19	No	chr11@ 105742772-105742795
GGAGACCAGCTCTCTCCTGG	CAG	0	5,18,19	No	chr11@ 24385014-24385037

Sequence	PAM	Similarity	Mism.	Gene	Locus
GGAGACCAGCTCTCTCCTGG	TGG	0	5,18,19	No	chr11@ 52991934-52991957
GGAGACCAGCTCTCTCCTGG	CAG	0	5,18,19	No	chr11@ 73916517-73916540
GGAGACCAGCTCTCTCCTGG	CGG	0	5,18,19	No	chr11@ 40482636-40482659
GGAGACCAGCTCTCTCCCAG	CGG	0	5,18,19	No	chr11@ 11919992-11920015
GGAGACCAGCTCTCTCCTGG	AGG	0	5,18,19	No	chr11@ 30637254-30637277
GGAGACCAGCTCTCTCCTGG	CAG	0	5,18,19	No	chr11@ 21620283-21620306
GGAGACCAGCTCTCTCCTGG	CAG	0	5,18,19	No	chr11@ 48573325-48573348
GGAGACCAGCTCTCTCCTGG	AGG	0	5,18,19	No	chr11@ 45601924-45601947
GGAGACCAGCTCTCTCCTGG	CAG	0	5,18,19	No	chr11@ 25552563-25552586
GGAGACCAGCTCTCTCCCAG	TGG	0	5,18,19	No	chr10@ 30563628-30563651
GGAGACCAGCTCTCTCCCAG	CAG	0	5,18,19	No	chr10@ 19305515-19305538
GGAGACCAGCTCTCTCCCAG	TGG	0	5,18,19	No	chr10@ 26225496-26225519
GGAGACCAGCTCTCTCCTAG	CAG	0	5,18,19	No	chr10@ 8034987-8035010
GGAGACCAGCTCTCTCCTAG	TGG	0	5,18,19	No	chr10@ 127771021-127771044
GGAGACCAGCTCTCTCCTGG	TGG	0	5,18,19	No	chr10@ 56169208-56169231
GGAGACCAGCTCTCTCCTGG	GAG	0	5,18,19	No	chr10@ 55096285-55096308
GGAGACCAGCTCTCTCCTGG	TGG	0	5,18,19	No	chr10@ 51052589-51052612
GGAGACCAGCTCTCTCCTGG	TGG	0	5,18,19	No	chr10@ 49693974-49693997
GGAGACCAGCTCTCTCCTGG	CGG	0	5,18,19	No	chr10@ 82720684-82720707
GGAGACCAGCTCTCTCCTGG	CAG	0	5,18,19	No	chr10@ 63251657-63251680
GGAGACCAGCTCTCTCCTGG	CGG	0	5,18,19	No	chr10@ 117271696-117271719
GGAGACCAGCTCTCTCCTGG	CAG	0	5,18,19	No	chr10@ 78610709-78610732
GGAGACCAGCTCTCTCCTGG	CAG	0	5,18,19	No	chr10@ 55481652-55481675
GGAGACCAGCTCTCTCCCTG	CAG	0	5,18,19	No	chr10@ 30592781-30592804
GGAGACCAGCTCTCTCCCAG	CGG	0	5,18,19	No	chr10@ 7876700-7876723
GGAGACCAGCTCTCTCCTGG	CAG	0	5,18,19	No	chr10@ 26623290-26623313
GGAGACCAGCTCTCTCCTGG	TGG	0	5,18,19	No	chr10@ 24425673-24425696
GGAGACCAGCTCTCTCCTGG	TGG	0	5,18,19	No	chr10@ 57098538-57098561
GGAGACCAGCTCTCTCCTGG	GAG	0	5,18,19	No	chr10@ 78766359-78766382
GGAGACCAGCTCTCTCCTGG	TGG	0	5,18,19	No	chr10@ 40611736-40611759
GGAGACCAGCTCTCTCCTGG	AGG	0	5,18,19	No	chr10@ 34422816-34422839
GGAGACCAGCTCTCTCCTGG	TGG	0	5,18,19	No	chr10@ 35817723-35817746
GGAGTCCAGCTCTCTCCTGG	TGG	0	5,18,19	No	chr10@ 53183548-53183571
GGAGACCAGCTCTCTCCCTG	TGG	0	5,18,19	No	chr9@ 89249417-89249440
GGAGACCAGCTCTCTCCTTG	TGG	0	5,18,19	No	chr9@ 87295227-87295250
GGAGACCAGCTCTCTCCTGG	TGG	0	5,18,19	No	chr9@ 84211635-84211658
GGAGACCAGCTCTCTCCTGG	CAG	0	5,18,19	No	chr9@ 118277981-118278004
GGAGTCCAGCTCTCTCCTGG	TGG	0	5,18,19	No	chr9@ 84213510-84213533
GGAGACCAGCTCTCTCCCAG	CAG	0	5,18,19	No	chr9@ 9549923-9549946
GGAGACCAGCTCTCTCCCGG	TGG	0	5,18,19	No	chr9@ 9550142-9550165
GGAGACCAGCTCTCTCCCGG	TGG	0	5,18,19	No	chr9@ 18367663-18367686
GGAGACCAGCTCTCTCCTTG	CAG	0	5,18,19	No	chr9@ 18057331-18057354
GGAGACCAGCTCTCTCCTGG	CAG	0	5,18,19	No	chr9@ 83912650-83912673

Sequence	PAM	Similarity	Mism.	Gene	Locus
GGAGACCAGCTCTCTCCTGG	CAG	0	5,18,19	No	chr9@ 71987023-71987046
GGAGACCAGCTCTCTCCTGG	CAG	0	5,18,19	No	chr9@ 5504810-5504833
GGAGTCCAGCTCTCTCCTGG	CAG	0	5,18,19	No	chr8@ 79102629-79102652
GGAGACCAGCTCTCTCCCAG	TGG	0	5,18,19	No	chr8@ 63402446-63402469
GGAGACCAGCTCTCTCCTGG	CAG	0	5,18,19	No	chr8@ 82606210-82606233
GGAGACCAGCTCTCTCCTGG	CAG	0	5,18,19	No	chr8@ 99140150-99140173
GGAGACCAGCTCTCTCCTGG	TGG	0	5,18,19	No	chr8@ 79814061-79814084
GGAGACCAGCTCTCTCCTGG	TGG	0	5,18,19	No	chr8@ 129130234-129130257
GGAGACCAGCTCTCTCCTGG	CAG	0	5,18,19	No	chr8@ 112526570-112526593
GGAGACCAGCTCTCTCCTGG	TAG	0	5,18,19	No	chr8@ 24854972-24854995
GGAGACCAGCTCTCTCCCAG	TGG	0	5,18,19	No	chr8@ 68713948-68713971
GGAGCCCAGCTCTCTCCCGG	AGG	0	5,18,19	No	chr7@ 74986073-74986096
GGAGACCAGCTCTCTCCCAG	CAG	0	5,18,19	No	chr7@ 137695058-137695081
GGAGACCAGCTCTCTCCCAG	TGG	0	5,18,19	No	chr7@ 108657955-108657978
GGAGACCAGCTCTCTCCTAG	TGG	0	5,18,19	No	chr7@ 24095846-24095869
GGAGACCAGCTCTCTCCTGG	CGG	0	5,18,19	No	chr7@ 18837862-18837885
GGAGACCAGCTCTCTCCTGG	CAG	0	5,18,19	No	chr7@ 120613658-120613681
GGAGACCAGCTCTCTCCCAG	CAG	0	5,18,19	No	chr7@ 123599964-123599987
GGAGACCAGCTCTCTCCTGG	TGG	0	5,18,19	No	chr7@ 26034252-26034275
GGAGACCAGCTCTCTCCTGG	CGG	0	5,18,19	No	chr7@ 36583380-36583403
GGAGACCAGCTCTCTCCTGG	TGG	0	5,18,19	No	chr7@ 103632109-103632132
GGAGACCAGCTCTCTCCTGG	TGG	0	5,18,19	No	chr7@ 8537273-8537296
GGAGACCAGCTCTCTCCTGG	TGG	0	5,18,19	No	chr7@ 9277276-9277299
GGAGACCAGCTCTCTCCTGG	TGG	0	5,18,19	No	chr7@ 7737689-7737712
GGAGACCAGCTCTCTCCTGG	TGG	0	5,18,19	No	chr7@ 26153959-26153982
GGAGACCAGCTCTCTCCCAG	CAG	0	5,18,19	No	chr6@ 43598617-43598640
GGAGACCAGCTCTCTCCTGG	CAG	0	5,18,19	No	chr6@ 104004073-104004096
GGAGACCAGCTCTCTCCTGG	TAG	0	5,18,19	No	chr6@ 73895275-73895298
GGAGACCAGCTCTCTCCTGG	CGG	0	5,18,19	No	chr6@ 43720895-43720918
GGAGACCAGCTCTCTCCTGG	CAG	0	5,18,19	No	chr6@ 57683611-57683634
GGAGACCAGCTCTCTCCTGG	TGG	0	5,18,19	No	chr6@ 73515541-73515564
GGAGACCAGCTCTCTCCTGG	TGG	0	5,18,19	No	chr6@ 59232325-59232348
GGAGACCAGCTCTCTCCTGG	AAG	0	5,18,19	No	chr6@ 111481497-111481520
GGAGTCCAGCTCTCTCCTGG	TGG	0	5,18,19	No	chr6@ 146088805-146088828
GGAGACCAGCTCTCTCCCAG	CAG	0	5,18,19	No	chr6@ 118543519-118543542
GGAGACCAGCTCTCTCCCGG	TGG	0	5,18,19	No	chr6@ 42232932-42232955
GGAGACCAGCTCTCTCCTGG	GGG	0	5,18,19	No	chr6@ 16240467-16240490
GGAGACCAGCTCTCTCCTGG	TGG	0	5,18,19	No	chr6@ 75992631-75992654
GGAGACCAGCTCTCTCCTGG	AGG	0	5,18,19	No	chr6@ 142244747-142244770
GGAGACCAGCTCTCTCCTGG	CGG	0	5,18,19	No	chr6@ 76394312-76394335
GGAGACCAGCTCTCTCCTGG	TGG	0	5,18,19	No	chr6@ 58371848-58371871
GGAGACCAGCTCTCTCCTGG	TGG	0	5,18,19	No	chr6@ 57889905-57889928
GGAGACCAGCTCTCTCCTGG	TGG	0	5,18,19	No	chr6@ 80583348-80583371

Sequence	PAM	Similarity	Mism.	Gene	Locus
GGAGACCAGCTCTCTCCTGG	TGG	0	5,18,19	No	chr6@ 115113868-115113891
GGAGACCAGCTCTCTCCTGG	CAG	0	5,18,19	No	chr5@ 27536100-27536123
GGAGACCAGCTCTCTCCTGG	CGG	0	5,18,19	No	chr5@ 50813566-50813589
GGAGACCAGCTCTCTCCTGG	CAG	0	5,18,19	No	chr5@ 20505322-20505345
GGAGACCAGCTCTCTCCTGG	TGG	0	5,18,19	No	chr5@ 23157131-23157154
GGAGACCAGCTCTCTCCTGG	TAG	0	5,18,19	No	chr5@ 29539189-29539212
GGAGACCAGCTCTCTCCTGG	TGG	0	5,18,19	No	chr5@ 108913327-108913350
GGAGACCAGCTCTCTCCTGG	CGG	0	5,18,19	No	chr5@ 108262499-108262522
GGAGACCAGCTCTCTCCTGG	TGG	0	5,18,19	No	chr5@ 33172393-33172416
GGAGACCAGCTCTCTCCTGG	TGG	0	5,18,19	No	chr5@ 9789863-9789886
GGAGACCAGCTCTCTCCCAG	CAG	0	5,18,19	No	chr5@ 25031958-25031981
GGAGTCCAGCTCTCTCCTGG	TGG	0	5,18,19	No	chr4@ 89497448-89497471
GGAGACCAGCTCTCTCCTGG	CAG	0	5,18,19	No	chr4@ 69562796-69562819
GGAGACCAGCTCTCTCCTGG	AGG	0	5,18,19	No	chr4@ 95044939-95044962
GGAGACCAGCTCTCTCCTGG	TAG	0	5,18,19	No	chr4@ 72992283-72992306
GGAGACCAGCTCTCTCCTGG	CAG	0	5,18,19	No	chr4@ 99043147-99043170
GGAGACCAGCTCTCTCCTGG	CAG	0	5,18,19	No	chr4@ 111998568-111998591
GGAGCCCAGCTCTCTCCTGG	AGG	0	5,18,19	No	chr4@ 17381303-17381326
GGAGACCAGCTCTCTCCCAG	TGG	0	5,18,19	No	chr4@ 72994090-72994113
GGAGACCAGCTCTCTCCCAG	CGG	0	5,18,19	No	chr4@ 13071651-13071674
GGAGACCAGCTCTCTCCCAG	CAG	0	5,18,19	No	chr4@ 9164593-9164616
GGAGACCAGCTCTCTCCCGG	TGG	0	5,18,19	No	chr4@ 108645046-108645069
GGAGACCAGCTCTCTCCTGG	CAG	0	5,18,19	No	chr4@ 61861904-61861927
GGAGACCAGCTCTCTCCTGG	CAG	0	5,18,19	No	chr4@ 73579663-73579686
GGAGACCAGCTCTCTCCTGG	TGG	0	5,18,19	No	chr4@ 30199658-30199681
GGAGACCAGCTCTCTCCTGG	TGG	0	5,18,19	No	chr4@ 151680874-151680897
GGAGACCAGCTCTCTCCTGG	TGG	0	5,18,19	No	chr4@ 108727823-108727846
GGAGACCAGCTCTCTCCTGG	CAG	0	5,18,19	No	chr4@ 62078377-62078400
GGAGACCAGCTCTCTCCTGG	CAG	0	5,18,19	No	chr4@ 62123282-62123305
GGAGACCAGCTCTCTCCTGG	TGG	0	5,18,19	No	chr4@ 83065235-83065258
GGAGACCAGCTCTCTCCTGG	TGG	0	5,18,19	No	chr3@ 159219907-159219930
GGAGACCAGCTCTCTCCTGG	AGG	0	5,18,19	No	chr3@ 4096360-4096383
GGAGACCAGCTCTCTCCTGG	TGG	0	5,18,19	No	chr3@ 124378684-124378707
GGAGACCAGCTCTCTCCTGG	CAG	0	5,18,19	No	chr3@ 26738833-26738856
GGAGACCAGCTCTCTCCTGG	CAG	0	5,18,19	No	chr3@ 34345807-34345830
GGAGACCAGCTCTCTCCTGG	TGG	0	5,18,19	No	chr3@ 32111198-32111221
GGAGACCAGCTCTCTCCTGG	CAG	0	5,18,19	No	chr3@ 114254410-114254433
GGAGACCAGCTCTCTCCTGG	CAG	0	5,18,19	No	chr3@ 113172052-113172075
GGAGACCAGCTCTCTCCTGG	AAG	0	5,18,19	No	chr3@ 57203611-57203634
GGAGACCAGCTCTCTCCTGG	CAG	0	5,18,19	No	chr3@ 69377100-69377123
GGAGACCAGCTCTCTCCTGG	TGG	0	5,18,19	No	chr3@ 5558766-5558789
GGAGACCAGCTCTCTCCTGG	TAG	0	5,18,19	No	chr3@ 11527095-11527118
GGAGACCAGCTCTCTCCTGG	CGG	0	5,18,19	No	chr3@ 46611273-46611296

Sequence	PAM	Similarity	Mism.	Gene	Locus
GGAGACCAGCTCTCTCCCAG	TGG	0	5,18,19	No	chr3@ 144908561-144908584
GGAGACCAGCTCTCTCCCAG	CAG	0	5,18,19	No	chr3@ 36306073-36306096
GGAGACCAGCTCTCTCCTTG	TGG	0	5,18,19	No	chr3@ 41652069-41652092
GGAGACCAGCTCTCTCCTTG	GGG	0	5,18,19	No	chr3@ 115173392-115173415
GGAGACCAGCTCTCTCCTTG	CAG	0	5,18,19	No	chr3@ 133700182-133700205
GGAGACCAGCTCTCTCCTGG	TGG	0	5,18,19	No	chr3@ 20346501-20346524
GGAGACCAGCTCTCTCCTGG	CAG	0	5,18,19	No	chr3@ 100015166-100015189
GGAGACCAGCTCTCTCCTGG	TGG	0	5,18,19	No	chr3@ 46919735-46919758
GGAGACCAGCTCTCTCCTGG	TGG	0	5,18,19	No	chr3@ 151512818-151512841
GGAGACCAGCTCTCTCCTGG	AGG	0	5,18,19	No	chr3@ 108746856-108746879
GGAGACCAGCTCTCTCCTGG	TGG	0	5,18,19	No	chr3@ 26868257-26868280
GGAGACCAGCTCTCTCCTGG	TGG	0	5,18,19	No	chr3@ 93119966-93119989
GGAGACCAGCTCTCTCCTGG	TGG	0	5,18,19	No	chr3@ 21381191-21381214
GGAGACCAGCTCTCTCCTGG	TGG	0	5,18,19	No	chr3@ 47072311-47072334
GGAGACCAGCTCTCTCCCGG	CAG	0	5,18,19	No	chr3@ 83121395-83121418
GGAGACCAGCTCTCTCCTAG	TAG	0	5,18,19	No	chr2@ 105869861-105869884
GGAGACCAGCTCTCTCCTGG	AGG	0	5,18,19	No	chr2@ 122903554-122903577
GGAGACCAGCTCTCTCCTGG	CAG	0	5,18,19	No	chr2@ 139063209-139063232
GGAGACCAGCTCTCTCCTGG	TAG	0	5,18,19	No	chr2@ 26689337-26689360
GGAGACCAGCTCTCTCCCAG	CAG	0	5,18,19	No	chr2@ 159197635-159197658
GGAGACCAGCTCTCTCCCAG	TGG	0	5,18,19	No	chr2@ 46195686-46195709
GGAGCCCAGCTCTCTCCTGG	CAG	0	5,18,19	No	chr2@ 17119860-17119883
GGAGACCAGCTCTCTCCCAG	CGG	0	5,18,19	No	chr2@ 159197142-159197165
GGAGACCAGCTCTCTCCCAG	TGG	0	5,18,19	No	chr2@ 60459507-60459530
GGAGACCAGCTCTCTCCCAG	TGG	0	5,18,19	No	chr2@ 35471154-35471177
GGAGACCAGCTCTCTCCTGG	AGG	0	5,18,19	No	chr2@ 172207843-172207866
GGAGACCAGCTCTCTCCTGG	TGG	0	5,18,19	No	chr2@ 83369849-83369872
GGAGACCAGCTCTCTCCTGG	CAG	0	5,18,19	No	chr2@ 49164550-49164573
GGAGACCAGCTCTCTCCTGG	CGG	0	5,18,19	No	chr2@ 150393519-150393542
GGAGACCAGCTCTCTCCTGG	AGG	0	5,18,19	No	chr2@ 17002933-17002956
GGAGACCAGCTCTCTCCTGG	TGG	0	5,18,19	No	chr2@ 54660679-54660702
GGAGACCAGCTCTCTCCTGG	TGG	0	5,18,19	No	chr2@ 17327529-17327552
GGAGACCAGCTCTCTCCTGG	TAG	0	5,18,19	No	chr2@ 126101331-126101354
GGAGACCAGCTCTCTCCTGG	CAG	0	5,18,19	No	chr2@ 5253245-5253268
GGAGACCAGCTCTCTCCCAG	TGG	0	5,18,19	No	chr1@ 127335569-127335592
GGAGACCAGCTCTCTCCCGG	TGG	0	5,18,19	No	chr1@ 4103047-4103070
GGAGACCAGCTCTCTCCCGG	CAG	0	5,18,19	No	chr1@ 88078854-88078877
GGAGACCAGCTCTCTCCCTG	CAG	0	5,18,19	No	chr1@ 17774490-17774513
GGAGACCAGCTCTCTCCTAG	CAG	0	5,18,19	No	chr1@ 106443078-106443101
GGAGACCAGCTCTCTCCTGG	CAG	0	5,18,19	No	chr1@ 76519730-76519753
GGAGACCAGCTCTCTCCTGG	AGG	0	5,18,19	No	chr1@ 172891985-172892008
GGAGACCAGCTCTCTCCTGG	TGG	0	5,18,19	No	chr1@ 78259010-78259033
GGAGACCAGCTCTCTCCTGG	CAG	0	5,18,19	No	chr1@ 34908583-34908606

Sequence	PAM	Similarity	Mism.	Gene	Locus
GGAGACCAGCTCTCTCCTGG	AGG	0	5,18,19	No	chr1@ 7533894-7533917
GGAGACCAGCTCTCTCCTGG	TGG	0	5,18,19	No	chr1@ 5630297-5630320
GGAGACCAGCTCTCTCCTGG	CAG	0	5,18,19	No	chr1@ 97487525-97487548
GGAGACCAGCTCTCTCCTGG	TAG	0	5,18,19	No	chr1@ 103930379-103930402
GGAGACCAGCTCTCTCCTGG	TGG	0	5,18,19	No	chr1@ 15757515-15757538
GGAGACCAGCTCTCTCCTGG	AGG	0	5,18,19	No	chr1@ 100157950-100157973
GGAGACCAGCTCTCTCCTGG	CAG	0	5,18,19	No	chr1@ 108158262-108158285
GGAGACCAGCTCTCTCCTGG	CAG	0	5,18,19	No	chr1@ 47353386-47353409
GGAGACCAGCTCTCTCCTGG	TGG	0	5,18,19	No	chr1@ 55552544-55552567
GGAGACCAGCTCTCTCCTGG	TGG	0	5,18,19	No	chr1@ 16756203-16756226
GGAGACCAGCTCTCTCCTGG	CAG	0	5,18,19	No	chr1@ 54208112-54208135
GGAGACCAGCTCTCTCCTGG	AGG	0	5,18,19	No	chr1@ 16860570-16860593
GGAGACCAGCTCTCTCCCTG	TGG	0	5,18,19	No	chr1@ 47231303-47231326
GGAGACCAGCTCTCTCCCAG	TAG	0	5,18,19	No	chr1@ 77571061-77571084
GGAGACCAGCTCTCTCCCAG	TGG	0	5,18,19	No	chr1@ 10823266-10823289
GGAGACCAGCTCTCTCCCAG	TGG	0	5,18,19	No	chr1@ 176157669-176157692
GGAGACCAGCTCTCTCCTTG	TGG	0	5,18,19	No	chr1@ 15862176-15862199
GGAGACCAGCTCTCTCCTAG	TGG	0	5,18,19	No	chr1@ 31428081-31428104
GGAGACCAGCTCTCTCCTAG	AGG	0	5,18,19	No	chr1@ 5675370-5675393
GGAGACCAGCTCTCTCCTAG	TGG	0	5,18,19	No	chr1@ 5549675-5549698
GGAGACCAGCTCTCTCCTGG	TGG	0	5,18,19	No	chr1@ 44244715-44244738
GGAGACCAGCTCTCTCCTGG	CAG	0	5,18,19	No	chr1@ 54631810-54631833
GGAGACCAGCTCTCTCCTGG	CAG	0	5,18,19	No	chr1@ 4233464-4233487
GGAGACCAGCTCTCTCCTGG	TAG	0	5,18,19	No	chr1@ 26258462-26258485
GGAGGCCAGATCTCTACACT	GAG	0	10,16,20	No	chr9@ 32416081-32416104
GGAGGCCAGCACTCTCTATG	TGG	0	11,17,19	No	chr6@ 53643668-53643691
GGAGGCTAGCTCTCTCCTCT	GAG	0	7,18,20	No	chrX@ 70666396-70666419
GGAGGCTAGCTCTCTCCTCT	GAG	0	7,18,20	No	chrX@ 132792325-132792348
GGAGGCTAGCTCTCTCCTCT	GAG	0	7,18,20	No	chrX@ 91197687-91197710
GGAGGCTAGCTCTCTCCTCT	GAG	0	7,18,20	No	chr18@ 59496434-59496457
GGAGGCTAGCTCTCTCCTCT	GAG	0	7,18,20	No	chr17@ 77301479-77301502
GGAGGCTAGCTCTCTCCTCT	GAG	0	7,18,20	No	chr16@ 61101473-61101496
GGAGGCTAGCTCTCTCCTCT	GAG	0	7,18,20	No	chr14@ 98241800-98241823
GGAGGCTAGCTCTCTCCTCT	GAG	0	7,18,20	No	chr14@ 97338402-97338425
GGAGGCTAGCTCTCTCCTCT	GAG	0	7,18,20	No	chr14@ 89331000-89331023
GGAGGCTAGCTCTCTCCTCT	GAG	0	7,18,20	No	chr13@ 106663917-106663940
GGAGGCTAGCTCTCTCCTCT	GAG	0	7,18,20	No	chr13@ 29237155-29237178
GGAGGCTAGCTCTCTCCTCT	GAG	0	7,18,20	No	chr10@ 70890076-70890099
GGAGGCTAGCTCTCTCCTCT	GAG	0	7,18,20	No	chr10@ 33314070-33314093
GGAGGCTAGCTCTCTCCTCT	GAG	0	7,18,20	No	chr6@ 109098957-109098980
GGAGGCTAGCTCTCTCCTCT	GAG	0	7,18,20	No	chr6@ 72036337-72036360
GGAGGCTAGCTCTCTCCTCT	GAG	0	7,18,20	No	chr6@ 132422192-132422215
GGAGGCTAGCTCTCTCCTCT	GAG	0	7,18,20	No	chr3@ 70052308-70052331

Sequence	PAM	Similarity	Mism.	Gene	Locus
GGAGGCTAGCTCTCTCCTCT	GAG	0	7,18,20	No	chr3@ 7483195-7483218
GGAGGCTAGCTCTCTCCTCT	GAG	0	7,18,20	No	chr3@ 47377717-47377740
GGAGGCAAGCTCTCTCCTCT	GAG	0	7,18,20	No	chr2@ 139439394-139439417
GGAGGCTAGCTCTCTCCTCT	GAG	0	7,18,20	No	chr2@ 63641606-63641629
GGAGGCTAGCTCTCTCCTCT	GAG	0	7,18,20	No	chr2@ 138845504-138845527
GGAGACCAGCTCTCTTCAGG	TGG	0	5,16,19	No	chr19@ 29757054-29757077
GGAGACCAGCTCTCTTCAGG	TGG	0	5,16,19	No	chr18@ 5906535-5906558
GGAGACCAGCTCTCTTCAGG	CAG	0	5,16,19	No	chr18@ 8255187-8255210
GGAGACCAGCTCTCTTCAGG	AGG	0	5,16,19	No	chr16@ 59858894-59858917
GGAGACCAGCTCTCTGCAGG	TGG	0	5,16,19	No	chr3@ 61572672-61572695
GCAGGCCAGCTCTGTCCATG	GGG	0	2,14,19	No	chr2@ 128085118-128085141
GGAGGCCAGTTCTCTCCTTG	CAG	0	10,18,19	No	chr11@ 57834333-57834356
GGAGGCCAGTTCTCTCCTGG	CAG	0	10,18,19	No	chr9@ 73701854-73701877
GGAGGCCAGGTCTCTCCTGG	CAG	0	10,18,19	No	chr5@ 23194590-23194613
GGAGCCCAGCTCTTTCCATG	AGG	0	5,14,19	No	chrX@ 81606549-81606572
GGAGACCAGCTCTTTCCAGG	CAG	0	5,14,19	No	chr10@ 27837442-27837465
GGAGACCAGCTCTGTCCAGG	CAG	0	5,14,19	No	chr10@ 90662942-90662965
GGAGACCAGCTCTGTCCAGG	TGG	0	5,14,19	No	chr10@ 47380065-47380088
GGAGACCAGCTCTTTCCAGG	TGG	0	5,14,19	No	chr9@ 14434569-14434592
GGAGACCAGCTCTTTCCAAG	TGG	0	5,14,19	No	chr3@ 99890769-99890792
GGAGACCAGCTCTTTCCAGG	AGG	0	5,14,19	No	chr2@ 37096430-37096453
GGAGGTCAGCTCTCTTCACT	GAG	0	6,16,20	No	chr11@ 84396647-84396670
GGAGGGCAGCTCTCTCTGCG	CAG	0	6,17,18	No	chr8@ 119006123-119006146
GGAGGCCATCTCTCTTCACC	TGG	0	9,16,20	No	chr16@ 28834580-28834603
GGAGGCGAGCTCTCTCCTAG	CGG	0	7,18,19	No	chrX@ 85281240-85281263
GGAGGCAAGCTCTCTCCTGG	CAG	0	7,18,19	No	chrX@ 96785875-96785898
GGAGGCAAGCTCTCTCCTGG	CAG	0	7,18,19	No	chrX@ 95806571-95806594
GGAGGCTAGCTCTCTCCTGG	TGG	0	7,18,19	No	chr17@ 82983562-82983585
GGAGGCTAGCTCTCTCCCTG	CAG	0	7,18,19	No	chr14@ 46861414-46861437
GGAGGCTAGCTCTCTCCTGG	CGG	0	7,18,19	No	chr11@ 41847667-41847690
GGAGGTCAGCTCTCTCCTGG	CAG	0	6,18,19	No	chr16@ 23695473-23695496
GGAGGTCAGCTCTCTCCTGG	AGG	0	6,18,19	No	chr10@ 37122749-37122772
GGAGGACAGCTCTCTCCTTG	AGG	0	6,18,19	No	chr6@ 64089717-64089740
GGAGGTCAGCTCTCTCCTGG	CAG	0	6,18,19	No	chr4@ 37518990-37519013
GGAGGCAAGCTCTCTTCAGG	GAG	0	7,16,19	No	chr13@ 82221819-82221842
GGAGGCCATCTCTCTCCTGG	CAG	0	9,18,19	No	chrX@ 117033005-117033028
GGAGGCCAACTCTCTCCCGG	TAG	0	9,18,19	No	chr1@ 10415719-10415742
GGAGGCCGGCTCTGGCCACG	TGG	0	8,14,15	No	chr14@ 51282439-51282462
GGAGACCAGCTCTCTTCCCG	TGG	0	5,16,18	No	chr13@ 92069186-92069209
GGAGGCAAGCTCTGTCCAGG	CAG	0	7,14,19	No	chr7@ 107995743-107995766
GGAGGCTAGCTCTTTCCAGG	CAG	0	7,14,19	No	chr3@ 10504161-10504184
GGAGACCAGCTCTTTCCTCG	TGG	0	5,14,18	No	chr17@ 93275166-93275189
GGAGGCCAGCTATCTCCTGG	CAG	0	12,18,19	No	chr19@ 9322638-9322661

Tnfsf11-sg2

Sequence	PAM	Exon	Activity	Off-target
GCCATCTCTCCCACGTCCCG	GGG	1	58	92

Guide Details	
Cut site	chr14 [+78,308,026 : -78,308,026]
GC %	70
Activity	58
Off-target	92

OFF-TARGET ANALYSIS:

Mismatches	0	1	2	3	Total
Coding	0	0	0	3	3
Non-Coding	0	0	1	27	28

OFF-TARGET HITS:

Sequence	PAM	Similarity	Mism.	Gene	Locus
GCCCTCTCCCCCACGTCCCC	CAG	1	4,9,20	Yes	chr7@ 4836174-4836197
GCCAGCTCTGCCAGGTCCCG	AAG	0	5,10,14	Yes	chr11@ 102064626-102064649
GCCAGCTCTCCCACCTCCAG	CAG	0	5,15,19	Yes	chr6@ 125031822-125031845
CCCATCTCGCCCACGTCCCC	GGG	1	1,9,20	No	chr6@ 118335281-118335304
ACCATCTCTCCCACGTCCAT	CAG	0	1,19,20	No	chr15@ 103328649-103328672
GCCTTTTCTCCCACTTCCCG	CAG	0	4,6,15	No	chr17@ 34030421-34030444
CCCATCTCTCCCACATCCCA	CAG	0	1,15,20	No	chr1@ 48043227-48043250
GCCAGCTCTCCCACGTGCTG	CAG	0	5,17,19	No	chrX@ 137165367-137165390
GCCAACTCTCCCACCTCCCA	AAG	0	5,15,20	No	chr9@ 57331002-57331025
GCCAGCTCTCCCACTTCCCT	TAG	0	5,15,20	No	chr8@ 113518567-113518590
GCCAGCTCTCCCACCTGCCG	CAG	0	5,15,17	No	chr5@ 49788664-49788687
GCCATACCTCCCACTTCCCG	TGG	0	6,7,15	No	chr5@ 84540224-84540247
GCCATCTCTCCCACCTCACG	GGG	0	15,18	No	chr1@ 133548365-133548388
GGCATCTCTCCCACTTCCTG	GAG	0	2,15,19	No	chr8@ 13430493-13430516
GCCACCTCTCCCACCTCCAG	TGG	0	5,15,19	No	chr12@ 30124249-30124272
GCGATCTCACCCAAGTCCCG	GGG	0	3,9,14	No	chrX@ 105289388-105289411
GCCATCCCTCCCACCTCCCT	AGG	0	7,15,20	No	chr1@ 76141909-76141932
GCCAGCTCTCCCACGGCCCT	GGG	0	5,16,20	No	chr4@ 63137888-63137911

Sequence	PAM	Similarity	Mism.	Gene	Locus
GCCAGCTCTCCCAAGTCCCA	CAG	0	5,14,20	No	chr11@ 99272497-99272520
GCCAGCTCTCCCAGGTCCCT	CAG	0	5,14,20	No	chr10@ 41008104-41008127
GCCAACTCTCCCAGGTCCCC	TAG	0	5,14,20	No	chr9@ 73125106-73125129
GCCATCTCTCAAACTTCCCG	AAG	0	11,12,15	No	chr8@ 88393082-88393105
GCCATCTCTCCAACGTCCAT	GGG	0	12,19,20	No	chr6@ 34778065-34778088
GCCATCACTCCCACCTCCTG	CAG	0	7,15,19	No	chr7@ 49255346-49255369
GTCATCTCTCCCAACTCCCG	CAG	0	2,14,15	No	chr18@ 67741223-67741246
GCCATCTCTCCAACCTCCTG	TAG	0	12,15,19	No	chr2@ 116194052-116194075
GCCATCTCTCCCACCTTCCC	AAG	0	15,17,20	No	chr1@ 69109072-69109095
GCCATCTCTCTCACGCCCTG	CAG	0	11,16,19	No	chr19@ 6825810-6825833
GCCATCTCTCCAACGCCCGG	TAG	0	12,16,19	No	chr12@ 83018950-83018973

Tnfsf11-sg3

Sequence	PAM	Exon	Activity	Off-target
GAGCCAATCAGCCTCCAGGA	GGG	P	67	77

Guide Details	
Cut site	chr14 [+78,308,116 : -78,308,116]
GC %	60
Activity	67
Off-target	77

OFF-TARGET ANALYSIS:

Mismatches	0	1	2	3	Total
Coding	0	0	0	0	0
Non-Coding	0	0	1	32	33

OFF-TARGET HITS:

Sequence	PAM	Similarity	Mism.	Gene	Locus
CAGCCAATCAGCGTCCAGGA	CAG	4	1,13	No	chr7@ 52576531-52576554
TTGCCAAACAGCCTCCAGGA	AGG	3	1,2,8	No	chr13@ 78227477-78227500
GACCAAAGCAGCCTCCAGGA	AGG	2	3,5,8	No	chr4@ 11676087-11676110
GAAGGAATCAGCCTCCAGGA	CAG	2	3,4,5	No	chr11@ 77740518-77740541
GCTCCCATCAGCCTCCAGGA	CAG	1	2,3,6	No	chr4@ 124624488-124624511
GAGAAAATCAACCTCCAGGA	AAG	1	4,5,11	No	chr3@ 92534116-92534139
CAGCCAAACAGACTCCAGGA	GAG	1	1,8,12	No	chr6@ 131455389-131455412
AAGCCAAGCAGCTTCCAGGA	AAG	1	1,8,13	No	chr15@ 54942196-54942219
GAGCCAAACCGTCTCCAGGA	AGG	1	8,10,12	No	chr17@ 69224111-69224134
GTGACAATCAGCCTCCAGCA	AAG	1	2,4,19	No	chr7@ 139751433-139751456
GAGACAAGCAGCATCCAGGA	GAG	1	4,8,13	No	chr6@ 81756928-81756951
TAGCCCCTCAGCCTCCAGGA	AAG	1	1,6,7	No	chr18@ 21894445-21894468
GAGACATTCAGCCTCCTGGA	TAG	1	4,7,17	No	chr2@ 66836841-66836864
GATCCAAGCAGCCTTCAGGA	GAG	1	3,8,15	No	chr6@ 125518531-125518554
GAGACAAACAGCCTGCAGGA	AGG	1	4,8,15	No	chr17@ 32080924-32080947
GAGCCCACCAGGCTCCAGGA	AGG	1	6,8,12	No	chr9@ 114553929-114553952
GAACCCATCAGCCTCCAGCA	CGG	1	3,6,19	No	chr7@ 46246433-46246456
GAGCCCCTGAGCCTCCAGGA	AGG	1	6,7,9	No	chr5@ 105560828-105560851
GACCCACTCAGCCTACAGGA	GGG	1	3,7,15	No	chr6@ 34281356-34281379
GTGCCAATCAGGCTCCAGCA	AGG	1	2,12,19	No	chr14@ 24760455-24760478

Sequence	PAM	Similarity	Mism.	Gene	Locus
GAGATAATCAGCCTCAAGGA	AGG	1	4,5,16	No	chr6@ 65926362-65926385
GAGCAAAGCAGCCTCAAGGA	TGG	0	5,8,16	No	chrX@ 71141650-71141673
GAGCCAATCTGTCTCCTGGA	AGG	0	10,12,17	No	chr2@ 150736290-150736313
AAGCCAATCAGCTTCCAGCA	TGG	0	1,13,19	No	chr7@ 123844813-123844836
GACCCAAGCAGCCCCCAGGA	TGG	0	3,8,14	No	chr5@ 130150526-130150549
GAGCTAATCACCCTTCAGGA	CAG	0	5,11,15	No	chr5@ 29898425-29898448
GAGCAACTCAGCCCCCAGGA	TGG	0	5,7,14	No	chr4@ 120869839-120869862
GAGCCAGTCAGCCTCCAGTT	GGG	0	7,19,20	No	chr9@ 34683131-34683154
GAGCCAATCAAGCTCCTGGA	AGG	0	11,12,17	No	chr17@ 44547438-44547461
GAGCCAAGCAGCCTGCAGCA	GAG	0	8,15,19	No	chr5@ 130466815-130466838
CAGCCAATCAGCTACCAGGA	AGG	0	1,13,14	No	chrX@ 129911572-129911595
GAGCCAATCAGCATGCAGAA	GGG	0	13,15,19	No	chr17@ 7782714-7782737
